# UV-Sensitive Photoreceptor Protein OPN5 in Humans and Mice

**DOI:** 10.1371/journal.pone.0026388

**Published:** 2011-10-17

**Authors:** Daisuke Kojima, Suguru Mori, Masaki Torii, Akimori Wada, Rika Morishita, Yoshitaka Fukada

**Affiliations:** 1 Department of Biophysics and Biochemistry, Graduate School of Science, The University of Tokyo, Bunkyo-Ku, Tokyo, Japan; 2 Japan Science and Technology Agency (JST), Precursory Research for Embryonic Science and Technology (PRESTO), Kawaguchi, Saitama, Japan; 3 Department of Organic Chemistry for Life Science, Kobe Pharmaceutical University, Kobe, Hyogo, Japan; 4 Department of Molecular Neurobiology, Institute for Developmental Research, Aichi Human Service Center, Kasugai, Aichi, Japan; Vanderbilt University, United States of America

## Abstract

A variety of animal species utilize the ultraviolet (UV) component of sunlight as their environmental cues, whereas physiological roles of UV photoreception in mammals, especially in human beings, remain open questions. Here we report that mouse neuropsin (OPN5) encoded by the *Opn5* gene exhibited an absorption maximum (λmax) at 380 nm when reconstituted with 11-*cis*-retinal. Upon UV-light illumination, OPN5 was converted to a blue-absorbing photoproduct (λmax 470 nm), which was stable in the dark and reverted to the UV-absorbing state by the subsequent orange light illumination, indicating its bistable nature. Human OPN5 also had an absorption maximum at 380 nm with spectral properties similar to mouse OPN5, revealing that OPN5 is the first and hitherto unknown human opsin with peak sensitivity in the UV region. OPN5 was capable of activating heterotrimeric G protein Gi in a UV-dependent manner. Immuno-blotting analyses of mouse tissue extracts identified the retina, the brain and, unexpectedly, the outer ears as the major sites of OPN5 expression. In the tissue sections of mice, OPN5 immuno-reactivities were detected in a subset of non-rod/non-cone retinal neurons as well as in the epidermal and muscle cells of the outer ears. Most of these OPN5-immuno-reactivities in mice were co-localized with positive signals for the alpha-subunit of Gi. These results demonstrate the first example of UV photoreceptor in human beings and strongly suggest that OPN5 triggers a UV-sensitive Gi-mediated signaling pathway in the mammalian tissues.

## Introduction

The ultraviolet (UV) component of sunlight is utilized in a variety of animal species for their environmental cues, *e.g.*, for flower discrimination and orientation/navigation in insects [1], [2] and for mate choice and parent-offspring communication in birds [3], [4]. Consistently, these animals have UV-sensitive photoreceptors in their eyes [5]. In mammals, some rodents such as mice have UV-sensitive cone photoreceptor cells in the retina [5], [6], whose function remains yet unknown, while most other mammalian species including human beings have been thought to lack such UV photoreception mechanisms [7].

Here we report that mammalian neuropsin (OPN5) encoded by the *Opn5* gene is a UV-sensitive photoreceptor. The *Opn5* gene was first identified in the mouse and human genomes, and its mRNA expression was detected in various neural tissues such as the brain, the spinal cord and the retina [8], though detailed expression pattern within these mammalian tissues has not been reported to date. The deduced amino acid sequence of OPN5 shows relatively low similarity (25–30%) to any other known opsins, indicating that this opsin forms a new subfamily in the rhodopsin family. The rhodopsin family consists of several subtypes, each of which binds either 11-*cis-* or all-*trans*-retinal as their chromophore [9]: The majority of the subtypes (*e.g.* rhodopsin) bind 11-*cis*-retinal and generally activate G proteins such as Gt (transducin), Gq, Go, and Gs. The others including retinochrome and RGR-opsin function as photoisomerases, which convert all-*trans*-retinal into 11-*cis* isomer in a light-dependent manner. Although the exon–intron structure of *Opn5* gene exhibits a similarity to the photoisomerase genes [8], OPN5 protein shows only weak sequence relationship to either type of opsins. Therefore, it remained unclear whether OPN5 functions as a GPCR-type photopigment or photoisomerase, or has functions other than those. Quite recently, avian homologues of OPN5 have been reported to function as a violet- (quail) or UV- (chicken) sensitive photopigment and to activate G protein signaling [10], [11]. These studies suggest spectral divergence of OPN5 among species, leaving a question as to whether mammalian OPN5 has the maximal sensitivity in the visible region like many other opsins, or in the UV region. The current study clearly determines the absorption maxima of mammalian OPN5 and is the first to report a UV photoreceptor protein in human beings. We also present several lines of evidence suggesting that OPN5 activates a Gi-mediated phototransduction pathway in the mammalian cells.

## Results

### Spectral properties of mouse and human OPN5

**Figure 1:**
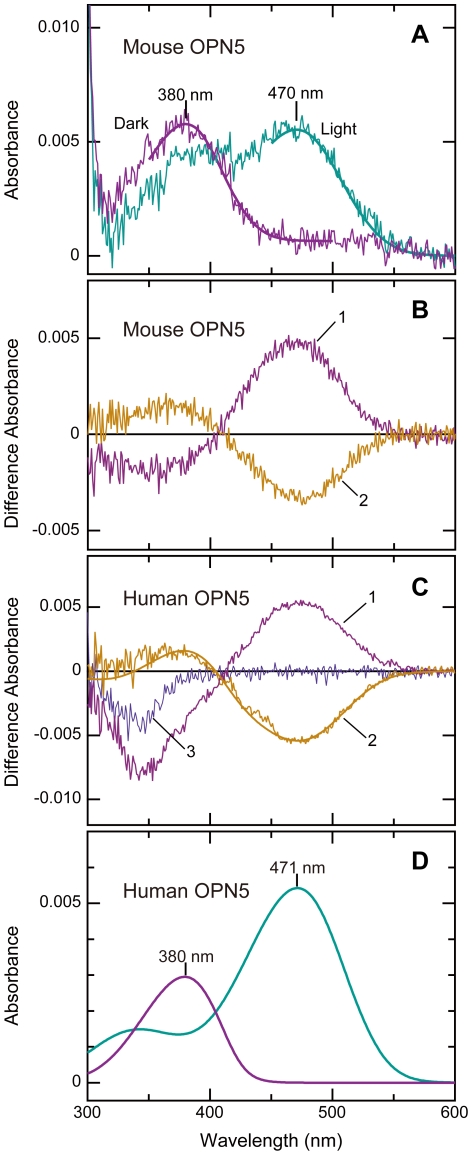
Mammalian OPN5 is a UV light-sensitive photopigment with 11-*cis*-retinal bound and exhibits a bistable nature. (A) Absorption spectra of mouse OPN5 (Dark) and its photoproduct (Light). The mouse OPN5 having 1D4-tag in the C-terminus was produced in a cell line of HEK293S cells (HEK293S-mOPN5#11), reconstituted with 11-*cis*-retinal, and affinity-purified with 1D4 antibody-immobilized resin (see Materials & Methods for details). The OPN5 (λmax 380 nm) was irradiated with UV light (357 nm; half band size, 10 nm; 28 µW/cm^2^) for 32 min, resulting in formation of a blue-absorbing photoproduct (λmax 470 nm). (B) Bistable photoreaction of mouse OPN5. The OPN5 in the dark (state 1; “Dark” in panel A) was first irradiated with 357-nm UV light (state 2; “Light” in panel A) and subsequently with >520-nm orange light (state 3; 10 mW/cm^2^, given that it was 550-nm monochromatic light). Shown are the difference spectra of state 2 minus state 1 (curve 1) and state 3 minus state 2 (curve 2), revealing back-and-forth photoreactions between OPN5 (λmax 380 nm) and the blue-absorbing photoproduct (λmax 470 nm). (C) Bistable photoreaction of human OPN5. The reconstituted sample for human OPN5 was obtained by solubilizing the membrane fraction of a stable cell line expressing human OPN5 (HEK293S-hOPN5#48). This partially purified human OPN5 was, as in panel B, subjected to illumination with 357-nm UV light (curve 1; 80 µW/cm^2^) and subsequently with >480-nm yellow light (curve 2; 3.6 mW/cm^2^, given that it was 550-nm monochromatic light) to obtain difference spectra in a similar manner to panel B. The difference spectra (curves 1 and 2) were similar in shape to those for mouse OPN5, indicating bistable photoreactions between a UV-absorbing pigment and a blue-absorbing species. Note that enhanced decrease of absorbance in <380-nm region during UV illumination (curve 1) can be partly due to photoreaction by non-OPN5 molecule(s) as this photoreaction also occurred in the control preparation using HEK293S cells (curve 3). (D) Estimation of the absorption spectrum for human OPN5. The spectra for human OPN5 and its photoproduct were estimated from curve 2 in panel C by using spectral templates for opsin-type photopigments (see Fig. S2 for details).

For functional analysis, we first attempted overexpression of mouse OPN5 in HEK293T cells by transient transfection without supply of retinals, which yielded no detectable amount of OPN5 protein. Then, we established a cell line of HEK293S cells stably expressing mouse OPN5 (HEK293S-mOPN5#11) with an 8-amino-acid 1D4 tag at the C-terminus. Supply of 11-*cis*-retinal to the culture medium of HEK293S-mOPN5#11 cells markedly increased the yield of OPN5 protein whereas addition of all-*trans*-retinal gained only a slight increase of the protein (Fig. S1A), suggesting preference of OPN5 protein for the 11-*cis* configuration of retinal chromophore over the all-*trans* form. The mouse OPN5 protein thus reconstituted with 11-*cis*-retinal was purified with an affinity column chromatography using 1D4 antibody. Spectroscopic measurement of purified OPN5 revealed its absorption spectrum peaking at 380 nm (Fig. 1A, Dark), indicating mouse OPN5 is a UV-sensitive photopigment.

Photoreaction of mouse OPN5 was examined spectroscopically by irradiating with UV light (357 nm): The spectrum of mouse OPN5 was shifted to longer wavelengths peaking at 470 nm (Fig. 1A, Light; Fig. 1B, curve 1). This blue-absorbing photoproduct was stable in the dark and reverted by the subsequent illumination with orange light (>520 nm) into the original UV-absorbing state (Fig. 1B, curve 2), suggesting reversible photoreactions between the two states. The spectral change induced by the UV light irradiation (Fig. 1B, curve 1) was almost symmetrical to that induced by the orange light (curve 2) with respect to the baseline. This result indicates the bistable nature of mouse OPN5. Repeated cycles of the alternate irradiations with the UV and orange light caused back-and-forth conversions between the UV- and blue-absorbing species (Fig. S2D), although it was accompanied by a gradual decrease in the absorbance.

We further established another cell line of HEK293S stably expressing human OPN5 (HEK293S-hOPN5#48). Addition of 11-*cis*-retinal to the culture medium significantly enhanced the protein yield, whereas all-*trans*-retinal was less effective as was observed for mouse OPN5 (Fig. S1B). Irradiation of human OPN5 with UV light (357 nm) decreased its absorbance in the UV region and increased it at longer wavelengths peaking at ∼470 nm (Fig. 1C, curve 1). A mirror-image spectral change (curve 2) was observed in the subsequent illumination by yellow light (>480 nm). Overall similarities of the spectrophotometric features between human OPN5 (Fig. 1C) and mouse OPN5 (Fig. 1B) suggested that mammalian OPN5 proteins share common properties such as the UV-sensitivity and its photo-convertibility to a blue-absorbing product. Based on the spectral changes, we estimated the absorption spectra of human OPN5 (λmax 380 nm) and its photoproduct (λmax 471 nm) by template fitting (Fig. 1D, Fig. S2). For the calculation, the spectral change induced by the yellow light irradiation (Fig. 1C, curve 2) was subjected to a series of template fittings for opsin-type photopigments [12], resulting in a “best-fit” difference spectrum (the smooth line superimposed on curve 2 in Fig. 1C). This method of calculation was validated by a similar template fitting for mouse OPN5: The estimated spectra of mouse OPN5 and its photoproduct (Fig. S2E–F) were well matched to the observed data for purified protein (Fig. 1A). Together, we propose that the *OPN5* gene encodes the hitherto unknown UV opsin in human beings.

### G protein activation by mouse OPN5

**Figure 2:**
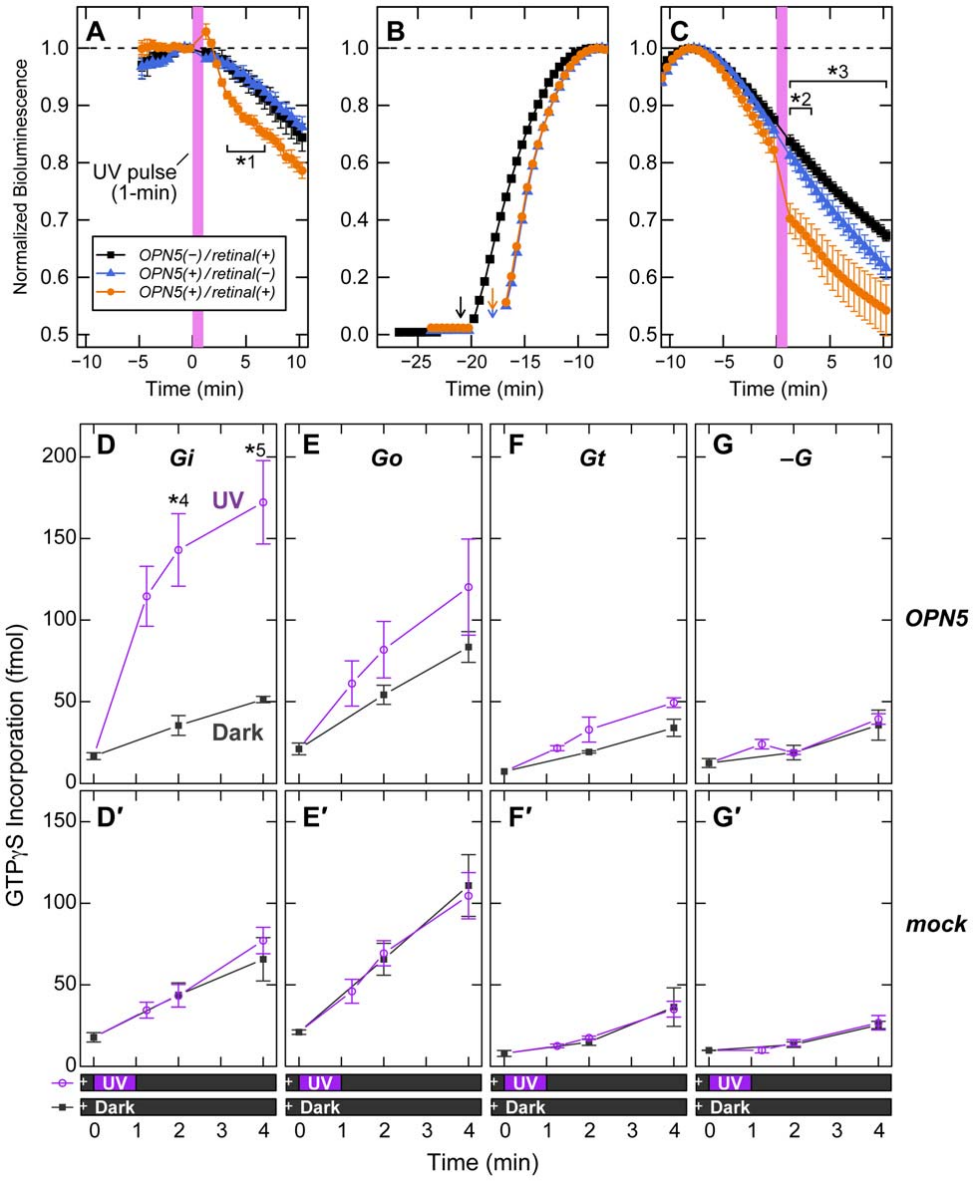
UV-dependent activation of Gi-type G protein by OPN5. (A–C) UV-induced cAMP reduction in OPN5-expressing HEK293S cells. The HEK293S-mOPN5#11 cells [*OPN5(+)*] or the wild-type HEK293S cells [*OPN5(–)*] were introduced with the expression vector of GloSensor protein, a luciferase derivative whose activity reflects cytosolic cAMP levels, and then the cells were cultured in the medium supplemented with 1 µM of 11-*cis*-retinal [*retinal(+)*] or without retinal [*retinal(–)*]. The bioluminescence of the cell culture was continuously monitored in the presence of luciferin. (A) The cells were first kept in the dark until their bioluminescence became stable, and then the cells were irradiated with 379-nm light for 1 min (indicated with *violet*). The bioluminescence values were normalized by those just before the irradiation for each dish. The data are represented by the mean ± SEM (*n* = 3) for *OPN5(–)/retinal(+)* and *OPN5(+)/retinal(+)*, or by the mean ± SD (*n* = 2) for *OPN5(+)/retinal(–)*. Statistical significance for the difference between the *OPN5(+)/retinal(+)* and *OPN5(–)/retinal(+)* data is shown as the asterisk (*1, *p*<0.05 by two-tailed Student's *t*-test). (B, C) Prior to the UV irradiation, the cells were stimulated with 10 µM of forskolin to increase the cAMP level. Shown in the panel B are the representative inductions in bioluminescence by forskolin, which was supplied into the cell culture at the indicated time points (*arrows*). Eight minutes after the bioluminescence peaking at the maximal values (C), the cells were irradiated with 379-nm light for 1 min (indicated with *violet*). The bioluminescence values were normalized by the maximal value for each dish. The UV-dependent reduction required both OPN5 and 11-*cis*-retinal. In the panel C, statistical significance of the *OPN5(+)/retinal(+)* data is shown as the asterisks (*2, *p*<0.05 against the *OPN5(+)/retinal(–)* data; *3, *p*<0.05 against the *OPN5(–)/retinal(+)* data) by one-way ANOVA with Tukey's post hoc test. (D–G, D′–G′) The UV-evoked activations of G proteins by OPN5 were measured by GTPγS-binding assays using the membrane fraction of the HEK293T/17 cells transfected with the expression construct for mouse OPN5 (D–G) or mock-transfected (D′–G′). Mixture of the membrane and the purified Gi (D, D′), Go (E, E′), Gt (F, F′), or no G protein (G, G′) was supplied with [^35^S] GTPγS (indicated by a *cross* in the bars under the horizontal axes) and then irradiated with 379-nm light (*UV*) or no light (*Dark*) for 1 min. The incorporated GTPγS was quantified at each time point after the irradiation and subjected to statistical analyses by two-tailed Student's *t*-test (*4, *p* = 0.0095; *5, *p* = 0.041). UV-dependent activation of Gi by OPN5 was detected in the panel D, whereas Go (E) and Gt (F) showed only slight tendencies of UV-dependent activation by OPN5, however, with no statistical significance at any time point. In the control experiments without OPN5 (*mock*), no significant difference in GTPγS incorporation was detected between *UV* and *Dark* (D′–G′). The data were represented by the mean ± SEM (*n* = 3; D–G, D′-F′) or by the mean ± SD (*n* = 2; G′).

We found that GTPγS-binding activity intrinsic to the membrane preparation from HEK293 cells was enhanced by UV irradiation in the presence of mouse OPN5 (Fig. S3). To identify the G protein subtype(s) activated by OPN5, we then examined an effect of UV illumination on the cAMP level in the HEK293S-mOPN5#11 cells into which a cAMP-sensing variant of luciferase (GloSensor, Promega) was introduced. In the first series of experiments, UV illumination onto the cells caused a slight but significant decrease in bioluminescent signal in an OPN5-dependent manner (Fig. 2A), suggesting UV-dependent inhibition of endogenous adenylate cyclase in the HEK293S cells. In order to enhance signal-to-noise ratio by increasing the bioluminescent signal to a semi-saturating level, forskolin was added to the cell culture for direct activation of endogenous adenylate cyclase in the second series of experiments (Fig. 2B). Subsequent UV illumination on the forskolin-activated cells consistently decreased the bioluminescent signal in the OPN5-expressing cells only when supplemented with 11-*cis*-retinal (Fig. 2C): The decrease in the bioluminescence signal during the UV illumination in the OPN5(+)/retinal(+) cells was statistically significant when compared with the cells of OPN5(–)/retinal(+) and OPN5(*+*)/retinal(–) (Fig. 2C). These data suggested that the cAMP level in the OPN5-expressing HEK293S cells is reduced by the UV irradiation probably via the endogenous Gi-signaling that inhibits adenylate cyclase activity.

We then examined whether mouse OPN5 can activate purified Gi/Go-class G proteins by using the OPN5-containing membrane preparation, of which intrinsic GTPγS binding activity had been eliminated by pre-treatment with GTPγS (Fig. 2G). When Gi was reconstituted with the OPN5-containing membranes, UV irradiation (at 379 nm) stimulated GTPγS incorporation to a significant degree (Fig. 2D), whereas no measurable increase was detected after the UV irradiation of mock HEK293 cells (Fig. 2D′). On the other hand, Go and Gt were only marginally activated in a UV-dependent manner even in the OPN5-containing membranes (Fig. 2E,F), although these G proteins were remarkably activated when higher concentration of Mg^2+^ ion was supplemented for Go (Fig. S4A) or light-activated rhodopsin was present for Gt (Fig. S4B). The results demonstrated that mouse OPN5 functions as a G protein-coupled receptor to selectively activate Gi-type G protein.

### Tissue and cellular localization of mouse OPN5

**Figure 3:**
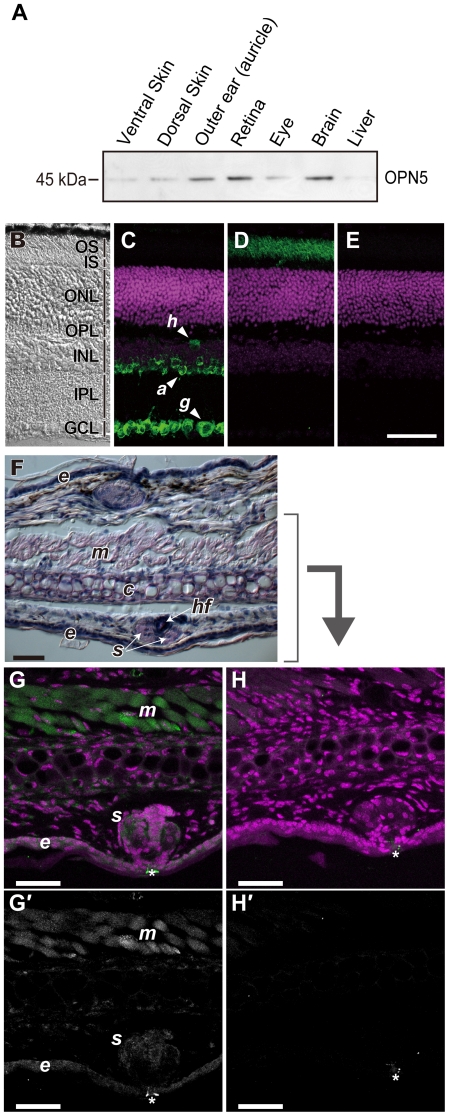
Localization of OPN5 protein in mouse tissues. (A) Western blot analysis of mouse tissue extracts by anti-OPN5 antibody. Ten microgram of total proteins were loaded in each lane for tissue extracts of ventral and dorsal skin, outer ears (auricles), retina, eyes, brain and liver. A stronger immuno-reactive signal was detected at 45 kDa in the outer ears (auricles), retina and brain. (B–E) Immunofluorescent examination of frozen sections from mouse retina. The sections were either imaged through differential interference contrast (B), or stained with anti-OPN5 antibody (C), with anti-Gαt1 antibody (D) or with depletion of primary antibody (E). (F–H) Histological and immunofluorescent examination of frozen sections from mouse outer ears (auricles); stained with hematoxylin-eosin (F), immuno-reacted with anti-OPN5 antibody (G and G′) or with rabbit normal IgG (H and H′). The panels C, D, E, G and H show immuno-reactive signals (*green*) as well as nuclear staining (*magenta*), while the panels G′ and H′ present the immuno-reactive signals alone (*white*) shown in the panels G and H, respectively. The signals indicated by asterisks (*) in the panels G, G′, H and H′ were autofluorescence of the hairs. *a*, amacrine cells; *g*, ganglion cells; *h*, horizontal cells; OS, outer segments of photoreceptors; IS, inner segments of photoreceptors; ONL, outer nuclear layer; OPL, outer plexiform layer; INL, inner nuclear layer; IPL, inner plexiform layer; GCL, ganglion cell layer. *c*, cartilage; *e*, epidermis; *hf*, hair follicle; *m*, striated muscle; *s*, sebaceous gland. Scale bars, 50 µm.

To characterize the tissue and cellular localization, we raised anti-mouse OPN5 antibody in rabbits. The immuno-purified antibody recognized 1D4-tagged OPN5 protein of 45 kDa (Fig. S6A), which is consistent with the calculated value (42.9 kDa). This band was also detected by 1D4 antibody (Fig. S6A), confirming the reactivity of the antibody to mouse OPN5. Western blot analysis of mouse tissue extracts using this antibody identified the brain, the retina and surprisingly the outer ears (auricles) as major sites for OPN5 protein expression (Fig. 3A and S6B). The protein expression pattern agreed well with the *Opn5* mRNA expression in the retina and the brain [8] as well as in the outer ears (Fig. S5). It should be emphasized that the 45 kDa OPN5 band was detected only in the detergent-soluble membrane fraction but not in the cytosolic fraction nor in the detergent-insoluble one of the tissue samples and also that no other band was detectable in these preparations (Fig. S6C and D). The results verified the specificity of the purified OPN5 antibody, which we used in the following immunohistological experiments of mouse tissues.

**Figure 4:**
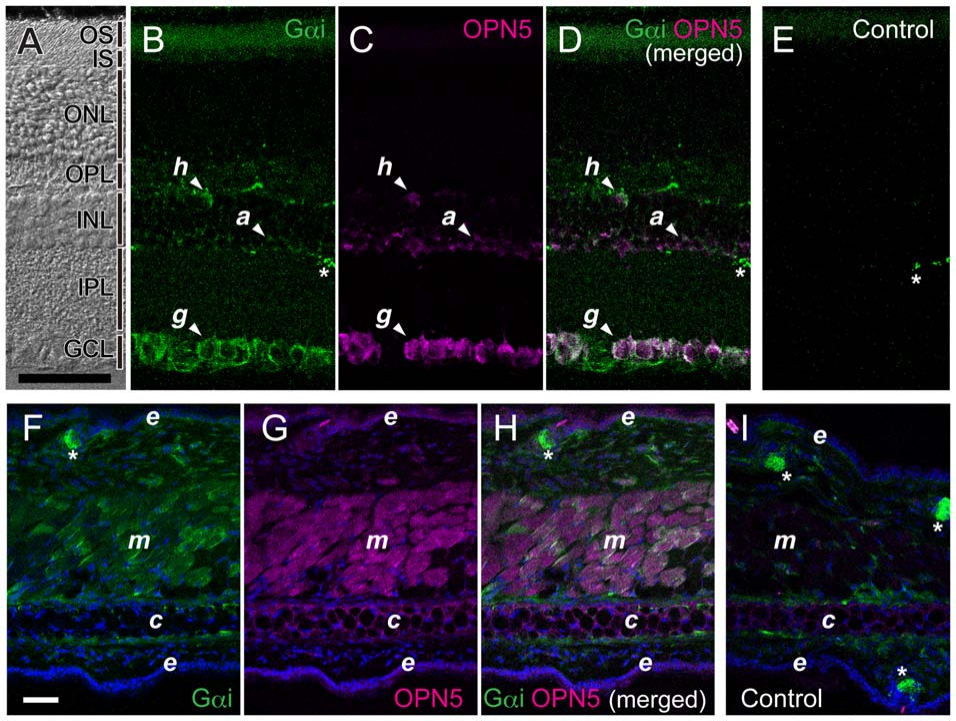
Co-localization of Gαi and OPN5 proteins in the retina and the outer ears of mice. (A–E) A section of mouse retina was reacted with antibodies to Gαi (sc-365422) and OPN5: The section was imaged through differential interference contrast (A), and subjected to fluorescence imaging for Gαi immuno-positive signals (B, *green*) and for OPN5 immuno-positive signals (C, *magenta*). The panel D shows the merged image of B and C, while the panel E gives its negative control (with depletion of the primary antibodies, *i.e.*, the secondary antibodies alone). (F–I) A section of mouse outer ear was reacted with antibodies to Gαi (sc-365422) and OPN5: The section was subjected to fluorescence imaging for Gαi immuno-positive signals (F, *green*) and for OPN5 immuno-positive signals (G, *magenta*). The panel H shows the merged image of F and G, while the panel I gives its negative control (with depletion of the primary antibodies, *i.e.*, the secondary antibodies alone). In the panels F–I, nuclear staining by TO-PRO-3 was colored in *blue*. Note that the *green* signals indicated by asterisks (*) in the panels B, D, E, F, H, and I originated from cross-reaction of the secondary antibody to the endogenous IgG present in the mouse tissues. Similar immuno-staining patterns of Gαi were detected by another antibody to Gαi [38] in the retina and the outer ears (see Fig. S9). Scale bars, 50 µm. Abbreviations are the same as in Figure 3.

Cellular localization of OPN5 protein in the retina was examined in the tissue sections (Fig. 3B–E). Strong signals for OPN5 immuno-reactivities were detected in a large number of retinal ganglion cells (Fig. 3C, *g*). Weaker but significant OPN5 signals were present in a subset of horizontal cells (*h*) and amacrine cells (*a*). No OPN5 signal was detectable in the photoreceptor layer (Fig. 3C), which was visualized by the antibody to rod transducin alpha subunit (Gαt1, Fig. 3D). These OPN5-immunoreactivities in the retinal neurons were mostly suppressed when the OPN5 antibody was preadsorbed with its antigenic peptide (Fig. S7B), confirming the specificity of the OPN5 antibody. The OPN5-positive signals in the retinal neurons were colocalized with Gαi immunoreactivities in major population (Fig. 4B–D). This observation, together with selective activation of Gi by UV-illuminated OPN5 (Fig. 2D), suggests that UV-activated OPN5 should trigger a Gi signaling pathway in these retinal neurons.

The strong OPN5-immuno-positive band in the lysate of the outer ears (Fig. 3A) led us to examine detailed expression pattern of OPN5 in the tissue sections (Fig. 3F–H). In the mouse outer ears (Fig. 3F), substantial signals of OPN5-immunoreactivities were detected in the striated muscle cells (*m*) and the weaker signals were observed in the epidermal cells (*e*) and the sebaceous gland cells (*s*). These OPN5-immunoreactivities in the outer ears were mostly suppressed by preadsorption of the OPN5 antibody with its antigenic peptide (Fig. S7E), confirming the specificity of the antibody to OPN5. Importantly, the OPN5-immunoreactive signals in the muscle and epidermal cells (Fig. 4G) were co-localized with the Gαi-positive signals (Fig. 4F and H). In the mouse ears, the muscle and epidermal cells should be the sites of OPN5-mediated UV-light reception leading to Gi signaling pathways.

## Discussion

In this study, we demonstrated that mouse and human OPN5 proteins are the photopigments with a peak sensitivity in the UV-region (Fig. 1). The mouse OPN5 can selectively activate Gi in a UV-dependent manner, thereby reducing cellular cAMP level possibly via inhibition of adenylate cyclase activity (Fig. 2). In mice, the OPN5 protein is present in a subset of non-rod/non-cone retinal neurons, as well as in the muscle and epidermal cells of the outer ears (Fig. 3). In most of these cells, OPN5 is co-localized with the alpha subunit of Gi (Fig. 4). These data suggest that mammalian OPN5 function as a UV-sensitive activator of Gi-mediated signaling pathways in these tissues.

Recently, two independent papers have reported the spectral properties of avian OPN5; *quail* OPN5 is violet-sensitive with the maximal sensitivity at around 420 nm [10], while *chicken* OPN5 is UV-sensitive having an absorption maximum at around 360 nm [11]. Such a large difference in the maximal sensitivity of OPN5 between the avian species raised a question as to whether mammalian OPN5s are violet- or UV-sensitive. The current study clearly demonstrates that both mouse and human OPN5s have the absorption maxima in the UV-A region (380 nm). OPN5 photopigments of other animal species could also be UV-sensitive if the amino acid sequences are similar to those of mammalian OPN5, though it needs extensive survey to be concluded.

**Figure 5:**
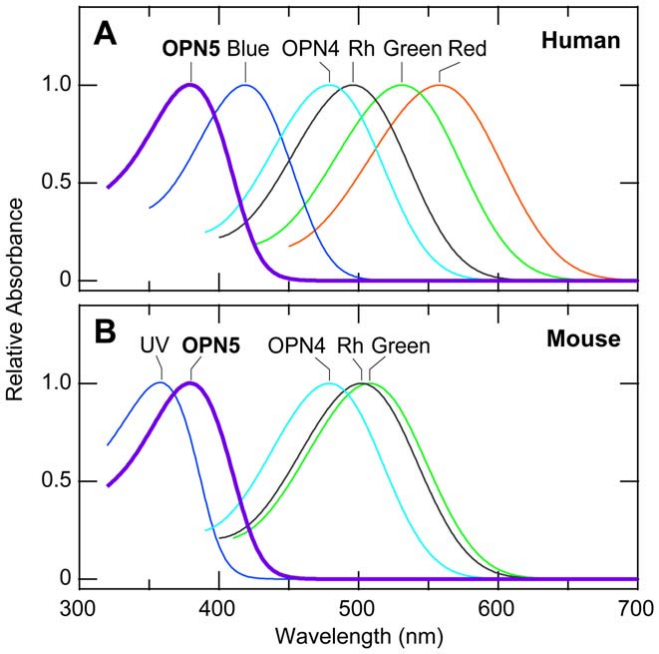
Schematic drawing of absorption spectra of opsin-type photopigments in human and mouse. These absorption spectra were drawn by using a spectral template [12] according to the reported values for absorption maxima (nm): human rhodopsin (Rh, 496 nm [40]), red (558 nm [40]), green (531 nm [40]), blue (419 nm [40]), OPN4 (479 nm [41]) , OPN5 (380 nm, this study); mouse rhodopsin (Rh, 502 nm [42]), green (508 nm [43]), UV (359 nm [44]), OPN4 (479 nm [45]), OPN5 (380 nm, this study).

To date, human beings have been thought to perceive the light only through the eyes. Rhodopsin and the three cone visual pigments (red, green and blue) are present in the retinal rod and cone photoreceptor cells, while OPN4 (melanopsin) is expressed in the intrinsically photosensitive retinal ganglion cells (ipRGC). These five known opsins have the absorption maxima between 400 and 560 nm, *i.e.*, in the visible region (Fig. 5), being consistent with the fact that the lenses of human eyes only transmit wavelength longer than 390 nm [13]. Even if human OPN5 is present in the retina, it would be unlikely to be activated effectively by light in the UV region. In contrast to an earlier study reporting the expression of *OPN5* mRNA in the human retina with no quantitative index [8], our quantitative PCR analysis of human *OPN5* mRNA detected no significant expression in the retina. Instead, non-retinal tissues such as the testis and its related tissue (epididymus) showed detectable levels of human *OPN5* mRNA expression. Testis expression was also reported for mouse *Opn5* mRNA [8]. A physiological role of *Opn5* expression in the testis should be investigated in future studies.

The retina was found to be one of the major expression sites for OPN5 protein in mice. In contrast to human beings, mice have UV-transmitting lenses in the eyes [14], allowing the UV-A to reach the retinal photoreceptor cells. Consistently, mice have UV-sensitive cones in the retina, and thus they show visual sensitivity to UV light, which can be significantly attenuated by loss of function in the cone photoreceptors [15]. In pupillary light reflex (PLR) measurement, UV-sensitivity remains almost intact in the *cone photoreceptor function loss* mutant [16], suggesting existence of another UV-sensitive system in the eyes. The UV sensitivity in PLR, however, is less likely attributable to OPN5 function because PLR to monochromatic UV light (360 nm) is severely diminished in the triple mutant mice that lack OPN4 and functional phototransduction mechanisms in rods and cones [17]. There has been another example of non-visual UV-sensitivity in mice: Retinal degenerate (*rd*) mutant mice, which gradually lose their rod and cone photoreceptor cells in their life, still show significant UV-sensitivity in light-induced phase shift of the circadian rhythm [18]. The UV-sensitivity in the phase-resetting of the circadian rhythm might involve OPN5 as a UV-sensitive photopigment in the mouse retina, though the role of OPN5 in the circadian photoreception could be limited, if any, because the sensitivity to white light in the circadian rhythm resetting is completely lost in the mutant mice lacking both OPN4 and functional rod/cone photoreceptors [17], [19].

OPN5 expression was unexpectedly detected in the outer ears of mice (Fig. 3). Because *Opn5* mRNA was previously detected in the mouse skin [20], we first examined OPN5 protein expression in the skin, but found it very faint (Fig. 3A). This observation should be reasonable because the trunk of mice is usually covered by pigmented hairs, which effectively cut off the UV content from the sunlight before reaching the skin. In contrast, the outer ears of mice have fewer hairs on their surface, which possibly enables the outer ears to perceive UV-light. What is the physiological role of OPN5 in the outer ears? Since mice have circadian clock in the peripheral tissues [21], we tested an effect of UV-A illumination on the circadian rhythm in the isolated outer ears of mice (Fig. S8): The cultured ears exhibited a clear circadian rhythm which was monitored by a bioluminescence reporter, but the UV-A illumination caused no significant effect on the bioluminescence rhythm. On the other hand, previous studies reported that UV-A irradiation induced immunological modulations in the mouse skins [22] including those of the outer ears [23]. UV irradiation on the human skin causes several acute responses such as erythema (sunburn) and tanning [24]; the latter includes “immediate pigment darkening”, whose action spectrum peaks in the UV-A region [25], [26]. These UV-A-induced phenomena might involve the OPN5 expressed in the outer ears, though it needs to be tested in future studies.

## Materials and Methods

### Animals

Animal experiments were conducted in accordance with guidelines set by The University of Tokyo and approved by the Committee on Animal Care and Use of the Graduate School of Science (The University of Tokyo) with the permit number 20-07. Wild-type mice having C57BL/6J background were purchased from Tokyo Laboratory Animals Science Co., Ltd. (Tokyo, Japan) and used in the following experiments unless otherwise stated. PER2::LUC mice [27] (B6.129S6-*Per2^tm1Jt^*/J) were purchased from The Jackson Laboratory (ME). The mice were housed at 23±1°C in cages with food and water available *ad libitum*, and they were entrained to 12-h light/12-h dark cycles for at least 7 days.

### Cell lines

Two variants from human embryonic kidney (HEK) 293 cells were used in this study: HEK293T/17 variant (ATCC CRL 11268) was used for transient transfection of cDNA as it constitutively expresses the simian virus 40 (SV40) large T antigen and has high transfectability [28]. On the other hand, HEK293S [29], which does not express the SV40 large T antigen, was used for establishing the derivative cell lines constitutively expressing OPN5 proteins (see below). These cells were grown in the D-MEM/F-12 (Invitrogen) with 10% fetal bovine serum (JRH) at 37°C in a 5% CO_2_ atmosphere.

### Mouse and human Opn5 cDNA

Full-length cDNA fragments of mouse *Opn5* were PCR-amplified from a mouse brain cDNA pool by using a pair of primers, 5′-AGCAT GGCCT TGAAC CACAC TGCCC-3′ (forward) and 5′-GATGG ACAGG TGCGG GGTTT CTCGG-3′ (reverse). The fragments were subcloned into pcDNA3.1/V5-His-TOPO (Invitrogen) or pCR2.1-TOPO (Invitrogen) and sequenced. These cDNA clones appeared to be derived from alternatively spliced variants encoded by the *Opn5* gene. Of these variants, only one transcript encoded the full-length protein as previously reported [8] and was thus used for further investigation, while all the other transcripts retained premature stop codons and thus encoded shorter proteins lacking the chromophore-binding lysine residue in the seventh transmembrane helix, indicating that they are unlikely to function as photoreceptors.

A full-length cDNA fragment of human *OPN5* was obtained from IMAGE Clone 8991910 (OpenBiosystems; Genbank# BC126194).

### Functional expression of OPN5 and its reconstitution with 11-cis-retinal

The mouse and human *OPN5* cDNAs encoding the full-length proteins were modified to have an additional 8-amino acid epitope sequence (ETSQVAPA) of anti-rhodopsin monoclonal antibody 1D4 [30] at its C-terminus, and subcloned into a mammalian expression vector, pcDNA3.1 (Invitrogen). In “transient” expression experiments, HEK293T/17 cells cultured in 10-cm diameter plates were transfected with 20 µg/plate of the OPN5 expression construct by the calcium-phosphate method as previously described [31]. Twenty-four hours after the transfection, the culture medium was exchanged to the one containing 5 µM of 11-*cis*- or all-*trans*-retinal for reconstitution in the culture. The cells were cultured for additional 24 h in the dark and harvested into buffer P [50 mM HEPES–NaOH, 140 mM NaCl, 1 mM dithiothreitol (DTT), 1 mM EDTA, 4 µg/mL aprotinin, 4 µg/mL leupeptin, 0.1 mM phenylmethylsulfonyl fluoride (PMSF), pH 6.5 at 4°C]. The harvested cells were pelleted by centrifugation and stored at −80°C until use.

### Cell lines stably expressing OPN5 proteins

To establish cell lines stably expressing OPN5 proteins, HEK293S cells cultured in a 10-cm diameter plate were transfected by a modified calcium-phosphate method [32] with 15 µg each of the expression constructs for OPN5. Three days after the transfection, the cells were passaged into 10 dishes and cultured for 10–14 days in a selection medium containing 0.75 mg/mL of G418 (Nakalai Tesque, Japan) to isolate the transformed cell lines. Here, 48 colonies were picked up and grown in 24-wells or 6-wells plates to be established as cell lines. To validate OPN5 expression in each cell line, a portion of the cells were cultured in the medium containing 1 µM of 11-*cis*-retinal for 24 h, and collected in buffer P. The collected cells were solubilized in 1% (w/v) *n*-dodecyl-β-d-maltoside (DM; Dojindo) in buffer P, and the solubilized proteins were subjected to an immunoblot analysis with 1D4 antibody. The cell line expressing OPN5 protein most abundantly, named HEK293S-mOPN5#11 (for mouse OPN5) or HEK293S-hOPN5#48 (for human OPN5), was used in the following experiments.

### Membrane preparation of OPN5-expressing cells

A membrane fraction of the OPN5-expressing cells was isolated by centrifugation flotation in a stepwise sucrose gradient according to the method previously described [33] with some modifications: Briefly, the cells were homogenized with a Polytron homogenizer in 8.6% (w/v) sucrose resolved in buffer P, and then layered on the top of 40% (w/v) sucrose in buffer P, followed by centrifugation in a swing bucket rotor (SW28, Beckman) at 110,000 ×*g* for 30 min. The membranes floating at the interface between 8.6% and 40% sucrose layers were collected and stored at −80°C until use.

### Immunoaffinity purification of the OPN5 pigment

The HEK293S-mOPN5#11 cell line was served for the start material to purify OPN5. In a typical experiment, the cells in 50 dishes (10-cm diameter) were cultured in the medium supplied with 1 µM of 11-*cis*-retinal for 24 h, and collected in buffer P. The membrane proteins were solubilized from the cells by addition of 1% (w/v) DM in buffer P with gently pipetting 50 times. The solubilized proteins were separated from the insoluble materials by centrifugation (125,000 ×*g*, 4°C, 30 min), and the resultant supernatant was gently mixed with the 1D4-immobilized resin by the rotation at 4°C overnight. Then, the resin was packed into a column (φ7 mm×15 mm), and washed with 500 µL of 0.5% DM in buffer P, followed by successive washes with 250 µL of 0.25%, 0.1% and 0.05% DM (w/v) in buffer P and with 2.5 mL of 0.02% (w/v) DM in buffer P. Then, OPN5 was eluted from the resin with 0.45 mg/mL of 1D4 epitope peptide (ETSQVAPA) dissolved in 0.02% DM (w/v) in buffer P.

### Immunoblot analyses

The samples were solubilized by being mixed with the 1/4 volume of SDS-sample buffer [50 mM Tris-HCl, 30% (v/v) glycerol, 10% sodium dodecyl sulfate (SDS), 250 mM DTT, 10 mM EDTA, 0.1% CBB R-250, pH 6.8 at 25°C]. The samples were then separated by SDS-polyacrylamide gel electrophoresis (SDS-PAGE, 10% acrylamide) and transferred to a polyvinylidene difluoride membrane (Millipore). The blot was pre-incubated for blocking with 1% (w/v) skim milk (BD Biosciences) in TBS [50 mM Tris–HCl, 200 mM NaCl, 1 mM MgCl_2_, pH 7.4 at 25°C] for one hour at 37°C. Then the blot was incubated with the primary antibody dissolved in the blocking solution at 4°C overnight or at 37°C for 1 h. The bound antibody was detected by horseradish peroxidase-conjugated anti-mouse IgG antibody or anti-rabbit IgG antibody (0.2 µg/mL, Kirkegaard & Perry Laboratories), in combination with an enhanced chemiluminescence detection system using Western Lightning Chemiluminescence Reagent (PerkinElmer Life Sciences) or ImmunoStar (Wako Pure Chemical Industries).

### Spectrophotometry

Reconstituted proteins were solubilized from the cells or their membrane fraction by the addition of 1% (w/v) DM in buffer P and by gently pipetting 50 times. The solubilized proteins were collected by centrifugation (125,000 ×*g*, 4°C, 30 min). Absorption spectra of the solubilized samples were recorded with a Shimadzu Model UV-2450 spectrophotometer. The sample was maintained at 0°C by a reflux condenser (Thermo NESLAB, model RTE-7 D1) and irradiated with a 1-kW tungsten halogen lamp (Master HILUX-HR, Rikagaku) or a 24-W metal halide lamp (LB-24, Solarc Lighting Technology). The wavelength of light was selected through a band-pass filter (UV35, Asahi spectra) or a cut-off filter (O-54, Y-50 or Y-47; Toshiba).

### Light intensity

Light intensities were measured by using an optical power meter (Model 3664, HIOKI) with an optical sensor (Model 9742-10, HIOKI) set at the positions of samples.

### Monitoring of cytosolic cAMP level

UV-induced reduction in cytosolic cAMP levels was measured by using GloSensor assay system (Promega). The cells of HEK293S-mOPN5#11 and HEK293S were plated on 35-mm dishes at 2.2×10^6^ cells/dish. In the next day of plating, the cells were transfected with 1.5 µg plasmid of pGloSensor-22F (Promega) by using Lipofectamine (Invitrogen) and PLUS reagent (Invitrogen). Twenty-four hours after the transfection, the medium was exchanged to the medium containing 1 µM of 11-*cis*-retinal for regeneration of OPN5, and the cells were further cultured for 24 hours in the dark. The following manipulations were performed in the dim red light (>640 nm) or in the dark. In the day of the GloSensor assay, the medium was exchanged to an assay medium [D-MEM (Sigma) with 10 mM of HEPES–NaOH, 4.5 g/L of glucose, 10% (v/v) fetal bovine serum, 25 units/mL of penicillin, 25 µg/mL of streptomycin, and 2 mM of luciferin, pH 7.0], and the cells were incubated at 25°C in the dark more than 2 hours for equilibrium. The bioluminescence from the cultured cells were measured by a luminometer (model TD-20/20, Turner Designs) every 30 sec with 25 sec of integration. In some experiments, the cells were supplied with forskolin (Calbiochem; resolved in DMSO) at the final concentration of 10 µM. The cells in the luminometer were irradiated with UV-light (379 nm, 22 µW/cm^2^) from the 24-W metal halide lamp for 1 min. The wavelength of light was selected through a band-pass filter (UV38, Asahi Spectra).

### GTPγS binding assay

G protein activation assays were carried out by measuring the amount of guanosine 5′-*O*-(3-thiotriphosphate) (GTPγS) bound to G proteins using the membrane fraction containing OPN5 described above. A small portion of the membrane fraction was solubilized in 1% (w/v) DM and spectrophotometrically analyzed to determine the OPN5 content, while the rest was used for the GTPγS binding reaction as follows. To eliminate the intrinsic GTPγS binding activity (see Figure S3), the membrane was treated with 5 µM of GTPγS at 4°C overnight in the pre-treatment buffer [20 mM Tris–HCl, 25 mM MgCl_2_, 250 mM (NH_4_)_2_SO_4_, 1 mM EDTA, 1 mM DTT, pH 7.5], and then washed with the buffer to remove excessive GTPγS. The membrane fraction was mixed with purified G protein (Go, Gi, or Gt) and then with [^35^S]GTPγS solution to start reaction. At 15 sec later, the sample was irradiated with 379 nm-light (53 µW/cm^2^) for 1 min (UV) or kept in the dark (Dark) at 4°C. Buffer composition of the reaction mixture was 10 mM MOPS–NaOH (pH 7.5 at 4°C), 30 mM NaCl, 60 mM KCl, 2 mM MgCl_2_, 0.195 mM CaCl_2_, 0.2 mM EGTA, 1 mM DTT, 4 µg/mL aprotinin, 4 µg/mL leupeptin, 0.5 µM [^35^S] GTPγS, 2 µM GDP, 0.043% CHAPS and 0.01% Lubrol-PX, 6.8×10^−3^ optical density unit (OD) of OPN5, 0.71 µg/µL of the solubilized HEK293S membrane protein, and 0.1 µM heterotrimeric G protein (Gi, Go, or Gt; see below). After the mixture was incubated for a selected amount of time in the dark at 4°C, its 10 µL aliquot was mixed with 100 µL of stop solution (20 mM Tris–HCl, 100 mM NaCl, 25 mM MgCl_2_, and 5 µM GTPγS, pH 7.4) and was immediately filtrated with nitrocellulose membrane (Millipore) to trap [^35^S] GTPγS bound to G proteins. The membrane was then washed four times with 200 µL of washing buffer (20 mM Tris–HCl, 100 mM NaCl, and 25 mM MgCl_2_, pH 7.4) to diminish free [^35^S] GTPγS, and put into 0.9 mL of scintillator (ACS II, GE Healthcare) to measure the radioactivity by a liquid scintillation counter. Gt (α and βγ subunits) was purified from bovine retina as described in literature [34], [35], while Go and Gi were mixtures of Goα [36] and Giα [37] with Gβγ from brain [36].

### Anti-mouse OPN5 antibody

To generate polyclonal antibody against mouse OPN5 protein, a synthetic peptide, CQDERLPHYLRDED, corresponding to amino acids 10–22 of mouse OPN5 (except for the first Cys residue of the peptide) was conjugated to keyhole limpet hemocyanin (Pierce) using *m*-maleimidobenzoyl-*N*-hydroxysulfosuccinimide ester (Sulfo-MBS; Pierce, IL) according to a protocol supplied by the manufacturer. Two rabbits were injected subcutaneously with the keyhole limpet hemocyanin-conjugated peptide emulsified in Freund's complete adjuvant (Difco). For the purification of the antibody, the peptide was conjugated to EAH-Sepharose (Amersham Biosciences) using Sulfo-MBS. Sera obtained from the immunized rabbits were applied to the peptide-coupled column, and the antibodies bound to the column were eluted with 0.1 M glycine (pH 2.7). The eluate was neutralized with a 0.1-fold volume of 1 M Tris–HCl (pH 8.0) and stored at 4°C.

### Protein extracts from mouse tissues

After maintained in 12-hr light/12-hr dark (LD) cycles at least for 1 week, 7-week-old male mice were sacrificed by cervical dislocation 6 h (Zeitgeber time 6, or ZT6) or 18 h (ZT18) after the onset of the light phase, and then tissues were dissected under room light (ZT6) or dim red light (>640 nm; ZT18). The tissues were homogenized at 4°C with the 10-fold volumes of buffer P10 (50 mM HEPES-NaOH, pH 6.5, 10 mM NaCl, 1 mM DTT, 3 mM MgCl_2_, 1 mM PMSF, 4 µg/ml aprotinin, 4 µg/ml leupeptin). The homogenate was centrifuged (19,000 ×*g*, 4°C, 15 min) to remove the supernatant, which was further centrifuged (125,000 ×*g*, 4°C, 60 min) to collect the supernatant (designated as *cytosolic fraction*). The precipitated pellet in the first centrifugation was then washed with buffer P10, and solubilized with 1% (w/v) DM in buffer P. The lysate was centrifuged (19,000 ×*g*, 4°C, 15 min) to collect the supernatant (designated as *tissue extract* or *detergent-solubilized fraction*) and the precipitated pellet (designated as *insoluble fraction*).

### Immunohistochemistry

Adult male mice were maintained in 12-hr light/12-hr dark (LD) cycles at least for 1 week, and anesthetized at ZT6 or ZT18 with 500 µL intraperitoneal injection of 6.5 mg/ml ketamine and 4.4 mg/mL xylazine, and then perfused with saline and subsequently with 4% paraformaldehyde in PBS (10 mM Na–phosphated buffer, 140 mM NaCl, 1 mM MgCl_2_, pH 7.4) under room light (ZT6) or dim red light (>640 nm, ZT18). Tissues were isolated from these animals, post-fixed in 4% paraformaldehyde in PBS overnight at 4°C, cryo-protected successively with 5, 10, 15 and 20% sucrose in phosphate buffer and embedded in a solution of 2∶1 mixture of 20% sucrose in PBS(−) (137 mM NaCl, 2.69 mM KCl, 5.5 mM Na_2_HPO_4_, 1.47 mM KH_2_PO_4_) and the OCT mounting medium (Sakura, Tokyo, Japan). The embedded tissues were frozen by using liquid nitrogen, and stored at −80°C until use. Finally, 10-µm-thick sections were cut out from the embedded tissues, mounted on MAS-coated glass slides (Matsunami Glass Ind., Japan), and air-dried.

The sections on the glass slides were rinsed with PBS, pretreated with a blocking solution [3% (v/v) goat normal serum, 0.1% (w/v) Triton X-100 in PBS] for 1 h at room temperature, and then incubated with a primary antibody diluted in the blocking solution at 4°C for 36 h (or at 25°C for 16 h). After rinsed with PBS, the sections were treated with a secondary antibody for overnight at 4°C and again washed with PBS. Then, the sections were coverslipped with Vectashield Mounting Medium (Vector Laboratories, Burlingame, CA). The primary antibodies used were rabbit anti-OPN5 antibody (described above; diluted to 0.16 µg/mL), a rabbit normal IgG (SIGMA-aldrich, MO; diluted to 0.16 µg/mL), rabbit anti-Gαt1 antibody (sc-389, Santa Cruz Biotechnology, CA; diluted to 0.011 µg/mL), mouse anti-Gαi antibody (sc-365422, Santa Cruz Biotechnology, CA; diluted to 4 µg/mL) and rabbit anti-Gαi antibody [38] (diluted to 0.2 µg/mL). The secondary antibodies used were goat anti-rabbit IgG antibody conjugated with Alexa488 and goat anti-mouse IgG antibody conjugated with Alexa568 (Invitrogen, CA; diluted to 2 µg/mL). TO-PRO-3 (Invitrogen, CA; diluted to 0.5 µM) was used for staining of cell nuclei.

### Real-time monitoring of PER2::LUC bioluminescence in the sliced outer ears

Adult PER2::LUC homozygous mice (9 or 10 wk-old) were maintained in 12-hr light/12-hr dark (LD) cycles at least for 1 week. Outer ears were collected from these mice, and sliced into ∼0.5 mm thickness with a stainless razor blade. Each of the slices was put on the Millicell Standing Insert (PICM0RG50; Millipore, MA) set in a 35-mm dish, which was filled with 1.3 mL of the recording medium [D-MEM (Sigma) with 10 mM of HEPES–NaOH, 4.5 g/L of glucose, 10% (v/v) fetal bovine serum, 25 units/mL of penicillin, 25 µg/mL of streptomycin, and 0.5 mM of luciferin, pH 7.0] and maintained at 37°C. Bioluminescence from the slice was continuously measured at 37°C in air with Dish Type Luminescencer, Kronos (AB-2500; ATTO, Tokyo, Japan) or LumiCycle (Actimetrics, IL) as previously described [39]. When cyclic oscillation of bioluminescence was observed, the highest or lowest level of the bioluminescence signals in each cycle was defined as the peak or trough, respectively. In this study, we defined the phase at the trough as circadian time (CT) 0, as the peak of bioluminescence oscillation has been reported to lie between CT12 and 16 in most tissues in PER2::LUC mice [27]. At a certain time point after the third trough of bioluminescence cycles was observed, the dish was irradiated with UV light from a 24-W metal halide lamp (LB-24, Solarc Lighting Technology). The wavelength of light was selected through a band-pass filter (UV35, Asahi spectra). In case of “dark pulse” controls, the dish was set in a light-tight steel can and irradiated in the same way as above.

## Supporting Information

Figure S1 **11-** ***cis*** **-retinal supplement during the culture increases the amount of recombinant OPN5 protein expressed in HEK293S cells.** HEK293S cells stably expressing 1D4-tagged mouse OPN5 (A, HEK293S-mOPN5#11) or human OPN5 (B, HEK293S-hOPN5#48) were cultured with 11-*cis* or all-*trans*-retinal supplemented. The cells were collected, solubilized with 1% DM in buffer P, and subjected to immunoblotting with the 1D4 antibody to detect the recombinant OPN5. Supplement with 11-*cis*-retinal to the cells (lanes 2) remarkably increased the amount of OPN5 protein expression in comparison with the sample without any retinal supplement (lane 1), while supplement with all-*trans*-retinal increased it with a much less degree (lane 3). The effect of all-*trans*-retinal supplement might be indirect as the parental HEK293S cells were reported to express retinoid cycle proteins [46].
(TIF)

Figure S2 **Inference of absorption spectrum of human OPN5.** (A) Spectral changes of recombinant human OPN5 during irradiations. HEK293S cells stably expressing human OPN5 (HEK293S-hOPN5#48) were cultured with 11-*cis*-retinal supplemented, and partially purified into OPN5-containing membrane by sucrose density gradient centrifugation. From this membrane sample, the OPN5 protein were solubilized with 1% DM in buffer P and subjected to spectral measurement. The OPN5 solution was first irradiated with 357-nm UV light (80 µW/cm^2^) for 4 min, and second with >480-nm yellow light (3.6 mW/cm^2^, given that it was 550-nm monochromatic light) for 4 min, both with a 24-W metal-halide lamp. Difference spectra were obtained by subtracting the spectrum measured at the first dark state from the one measured after UV irradiation (curve 1) and by subtracting the one after UV irradiation from the one after the yellow irradiation (curve 2). Similar spectral changes were obtained in subsequent repeated irradiations alternately with UV and yellow light (curves 3–12) with gradually decreasing amplitude. (B, C) Inference of human OPN5 spectrum. The difference spectrum between human OPN5 and its photoproduct (curves 2 in panel A and B) was used for the template fitting: First, 450–600 nm region of this difference spectrum (curve 2 in panel B) was best fit to a template for opsin-type photopigments [12] to infer the spectrum of OPN5 photoproduct (curve 2 in panel C). Second, the inferred photoproduct spectrum, peaking at 471 nm, was added to the curve 2 in panel B, to calculate the spectrum corresponding to the dark state OPN5. Finally, this dark state OPN5 spectrum (in the 350–500 nm region) was best fit to the spectral template of opsin-type photopigments, obtaining the curve 1 in panel C. A smooth curve in panel B (superimposed to curve 2) is the reconstructed difference spectrum between inferred OPN5 (curve 1 in panel C) and its photoproduct (curve 2 in panel C) to confirm the validity of the fitting. (D) Spectral changes of recombinant mouse OPN5 during irradiations. HEK293T/17 cells transiently expressing mouse OPN5, cultured with 11-*cis*-retinal supplemented, were used to prepare OPN5-containing membrane, from which the OPN5 protein sample was extracted. The OPN5 solution was irradiated with 357-nm UV light (28 µW/cm^2^) for 16 min and second with >520-nm orange light (10 mW/cm^2^, given that it was 550-nm monochromatic light) for 4 min from a 1-kw tungsten halogen lamp, resulting in the spectral changes shown as curves 1 and 2, respectively. Similar spectral changes were observed in subsequent irradiation with UV (curve 3) and orange (curve 4) light. (E, F) Inference of mouse OPN5 spectrum from the spectral change shown in panel D by the same method as in panel B and C. Note that the absorption maxima estimated by this method are quite similar to the ones obtained for absolute absorption spectra of purified mouse OPN5 and its photoproduct in Fig. 1A. (G) Spectral changes for the membrane extract for HEK293S cells having no OPN5 protein during repeated and alternated irradiation with UV (curves 1, 3, 5, 7, 9 and 11) and yellow light (curves 2, 4, 6, 8, 10 and 12) as in panel A. The absorbance decrease around 340 nm was observed during irradiation with UV, but not with yellow light.
(TIF)

Figure S3 **UV-dependent activation of G protein by OPN5.** The light-evoked activations of G proteins endogenously expressed in HEK293T/17 cells were measured by a GTPγS-binding assay. The OPN5-containing membrane was fractionated from the HEK293T/17 cells transfected with the expression construct for mouse OPN5 (or the empty vector). The prepared membrane was irradiated with UV-light (357 nm; 14 µW/cm^2^) for 1 min, and supplied with [S] GTPγS. The incorporated GTPγS during 15 sec of the reaction was quantified. The UV-dependent induction of GTPγS binding activity was detected only in the OPN5-containing membrane with a statistical significance (*p* = 0.024, two-tailed Student's *t*-test). Note that no exogenous G protein was supplied into the mixture, indicating the observed GTPγS binding stemmed from the intrinsic activity in the HEK293T/17 cells. All the data were represented by the mean ± SEM (*n* = 3).
(TIF)

Figure S4 **Mg^2+^- or rhodopsin-induced activity of the G proteins.** (A) Mg^2+^-induced intrinsic activity of Gi and Go. Reaction mixture containing Gi (*squares*), Go (*circles*) or no G protein (*diamonds*) was incubated with [^35^S] GTPγS in the presence of high concentration of Mg^2+^ ion at 30°C to measure the maximal activity of GTPγS binding. Buffer composition of the reaction mixture was 20 mM Tris-HCl (pH 7.5), 20 mM MgCl_2_, 250 mM (NH_4_)_2_SO_4_, 1 mM EDTA, 1 mM DTT, 0.01% Lubrol-PX, 2 µg/µL ovalbumin, 0.1 µM GDP, 1 µM [^35^S] GTPγS, 0.1 µM heterotrimeric G protein (Gi or Go). After the mixture was incubated for a selected amount of time, its aliquot (10 µL) was mixed with 100 µL of stop solution and immediately filtrated with nitrocellulose membrane (Millipore) to trap [^35^S] GTPγS bound to G proteins, as described in the Materials and Methods section. (B) GTPγS binding activity of Gt induced by photo-activated rhodopsin. The HEK293T/17 membrane containing bovine rhodopsin was prepared in a similar way for mouse OPN5. The membrane was mixed with Gt or no G protein and then irradiated with yellow light (>490 nm, *Light*) with the light intensity of 10 mW/cm^2^ (given that it was 550-nm monochromatic light), or kept in the dark (*Dark*) for 1 min. Buffer composition of reaction mixture was the same as that for Figure 2 except for absence of GDP in this experiment. The incorporated GTPγS was quantified at each time point. Data were represented by the mean ± SEM (*n* = 3).
(TIF)

Figure S5 ***Opn5*** **mRNA expression in mouse tissues examined by RT-PCR.** RNA from mouse tissues (ventral and dorsal skin, outer ear, retina, eye and liver) was subjected to RT-PCR experiment with (+RT) or without (–RT) reverse transcriptase. The 503-bp DNA fragment derived from *Opn5* mRNA (*arrowhead*) was only detected in “+RT” samples but not in “–RT” samples.
(TIF)

Figure S6 **Immunoblot analysis of OPN5 in the mouse tissue extracts.** (A) Examination of OPN5 antibody specificity. Loaded are the detergent-extract of membrane fraction of mouse retina (*retina*), mouse OPN5-expressing (*mOPN5*) and non-transfected HEK293S cells (*293S*). The membranes were reacted with the anti-OPN5 antibody (*left*) and with the 1D4 antibody (*right*). Both antibodies detected the 45 kDa-band (*solid arrowhead*) in *mOPN5*, demonstrating that the anti-OPN5 antibody recognizes the (1D4-epitope-tagged) OPN5 protein. In the mouse *retina*, the anti-OPN5 antibody detected a single band having a similar size (45 kDa), which should be the mouse native OPN5 protein. In the *mOPN5*, this antibody detected another band (36 kDa, *open arrowhead*), which is likely to be an endogenous protein of the human cell line (HEK293S) as it was also detected in the parent *293S* by the anti-OPN5 antibody. Importantly, the 36-kDa band was undetectable in any of the mouse tissues tested (see also B–D). (B) Tissue distribution pattern of OPN5 protein expression in mice. Loaded were the tissue extracts containing 10 µg of total proteins from ventral skin, dorsal skin, outer ear, retina, eye, brain, and liver. The blotted membrane was reacted with the anti-OPN5 antibody (*left*) to visualize OPN5 expression, or with depletion of the primary antibody (*right*). Note that only a single 45-kDa band (*closed arrowhead*) was detected with the anti-OPN5 antibody (*left*). As a loading control (B′), the proteins blotted on these membranes were visualized with MemCode Reversible Protein Stain Kit (Pierce/Thermo Scientific). (C, D) OPN5 localization in the tissue fractions from mouse retina (C) and outer ears (D). The *cytosolic*, detergent-*solubilized* and *insoluble* fractions from these tissues (see Materials and Methods for details) were loaded as well as the detergent-solubilized membrane fraction of mouse OPN5-expressing HEK293S cells (*mOPN5*, as a positive control). The blotted membrane was reacted with the anti-OPN5 antibody (*left*) or with depletion of the primary antibody (*right*). Note that the 45-kDa OPN5 protein was detected only in the *solubilized* among the three fractions and that no other band was detectable in either of the three fractions. These data verified the specificity of the anti-OPN5 antibody in the mouse tissues.
(TIF)

Figure S7 **Peptide-preadsorption experiments to examine specificity of immunofluorecent detection of OPN5 in the frozen sections from mouse retina (A–C) and outer ears (D–F).** The sections were immuno-reacted with the OPN5 antibody (A, D), or with the antibody that was pre-incubated with ten-fold molar excess of its antigenic peptide (B, E). The sections in C and F were immuno-reacted with depletion of the primary antibody (the secondary antibody alone). Immuno-reactive signals (*green*) are shown with nuclear staining by TO-PRO-3 (*magenta*). The peptide-preadsorption treatment (B, E) strongly suppressed the immuno-reactive signals in the ganglion cells (*g*), the amacrine cells (*a*) and the horizontal cells (*h*) in the retina as well as those in the epidermis (*e*), the striated muscle (*m*) and the sebaceous gland (*s*) in the outer ears. Note that in the outer ears (D–F), the hairs (*) exhibit autofluorescence while cartilage (*c*) has non-specific binding of the secondary antibody. ONL, outer nuclear layer; OPL, outer plexiform layer; INL, inner nuclear layer; IPL, inner plexiform layer; GCL, ganglion cell layer. Scale bars, 50 µm.
(TIF)

Figure S8 **UV irradiation on isolated outer ears of PER2::LUC mice causes no detectable effect on circadian expression of PER2::LUC bioluminescence.** (A) Plot of PER2::LUC bioluminescence from a cultured slice of outer ear, showing a circadian rhythm. The sample was subjected to a 30-min pulse of UV light (*UV pulse*; 357 nm, 11 µW/cm^2^) from 67.5 h after the initiation of the bioluminescence measurement. This time point (time of stimuli) was calculated as CT 1.99 according to (i) an estimated period for the bioluminescence cycles *before* the UV pulse and (ii) the third trough as a “datum point” (CT 0). In a similar way, the same time point was calculated as CT 2.05 according to (i) an estimated period for the bioluminescence cycles *after* the UV pulse and (ii) the fourth trough as a “datum point” (CT 0). From these two different estimates, the phase shift induced by the UV pulse was calculated as 0.06 h in this panel. (B) Plot of PER2::LUC bioluminescence from another cultured slice of outer ear, which was subjected to a 30-min pulse of dark incubation (*dark pulse*) as a control. (C) Plot of the phase shifts induced by UV-pulse (*open circles*) and dark-pulse (*closed triangles*) treatments on the outer ear slices against the time of stimulus. No obvious difference was observed between the phase-shifting effects of the UV-pulse and the dark-pulse treatments.
(TIF)

Figure S9 **Immunofluorescent examination by anti-Gαi antibodies of the frozen sections from mouse retina (A–E) and outer ears (F–J).** The sections were immuno-reacted with mouse anti-Gαi antibody (sc-365422) (B, G), rabbit anti-Gαi antibody [38] (C, H) and rabbit anti-OPN5 antibody (D, I). The sections reacted with the secondary antibody alone (*i.e.*, with depletion of the primary antibody) are shown in A and F as the negative controls for B and G (mouse antibody), and shown in E and J as the negative controls for C–D and H–I (rabbit antibodies). The immuno-reactive signals (*green*) are shown with nuclear staining by TO-PRO-3 (*magenta*). In the retinal sections, the immuno-reactive signals were detectable in the horizontal (*h*), amacrine (*a*) and ganglion (*g*) cells by the mouse and the rabbit anti-Gαi antibodies (B, C) as well as the anti-OPN5 antibody (D). In the outer ear sections, the immuno-reactive signals were detectable in the striated muscle cells (*m*) by the mouse and the rabbit anti-Gαi antibodies (G, H) as well as the anti-OPN5 antibody (I), while the signals in the epidermal cells (*e*) largely varied in strength among these antibodies. Note that the *green* signals indicated by asterisks (*) in the panels A, B, G and H originated from direct reaction of the secondary antibody to mouse IgG. OS, outer segments of photoreceptors; ONL, outer nuclear layer; OPL, outer plexiform layer; INL, inner nuclear layer; IPL, inner plexiform layer; GCL, ganglion cell layer; *c*, cartilage.
(TIF)
