# Supplementary figures and images for: UV-Sensitive Photoreceptor Protein OPN5 in Humans and Mice
Source: PLoS One. 2011 Oct 17;6(10):e26388. doi: 10.1371/journal.pone.0026388 (PMC3197025; doi:10.1371/journal.pone.0026388)

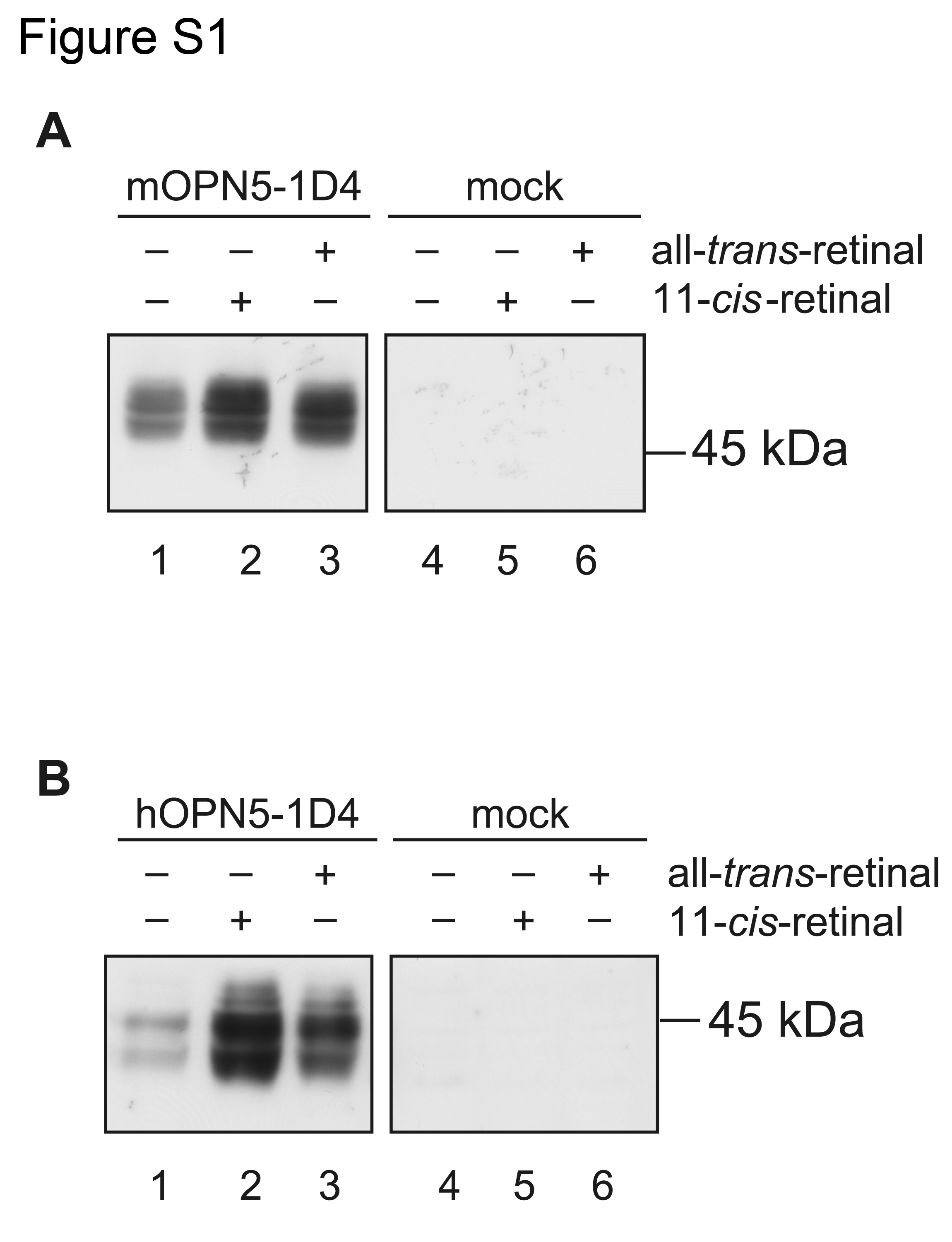

Supplement: Figure S1 — 11- cis -retinal supplement during the culture increases the amount of recombinant OPN5 protein expressed in HEK293S cells. HEK293S cells stably expressing 1D4-tagged mouse OPN5 (A, HEK293S-mOPN5#11) or human OPN5 (B, HEK293S-hOPN5#48) were cultured with 11-cis or all-trans-retinal supplemented. The cells were collected, solubilized with 1% DM in buffer P, and subjected to immunoblotting with the 1D4 antibody to detect the recombinant OPN5. Supplement with 11-cis-retinal to the cells (lanes 2) remarkably increased the amount of OPN5 protein expression in comparison with the sample without any retinal supplement (lane 1), while supplement with all-trans-retinal increased it with a much less degree (lane 3). The effect of all-trans-retinal supplement might be indirect as the parental HEK293S cells were reported to express retinoid cycle proteins [46]. (TIF) [file pone.0026388.s001.tif]

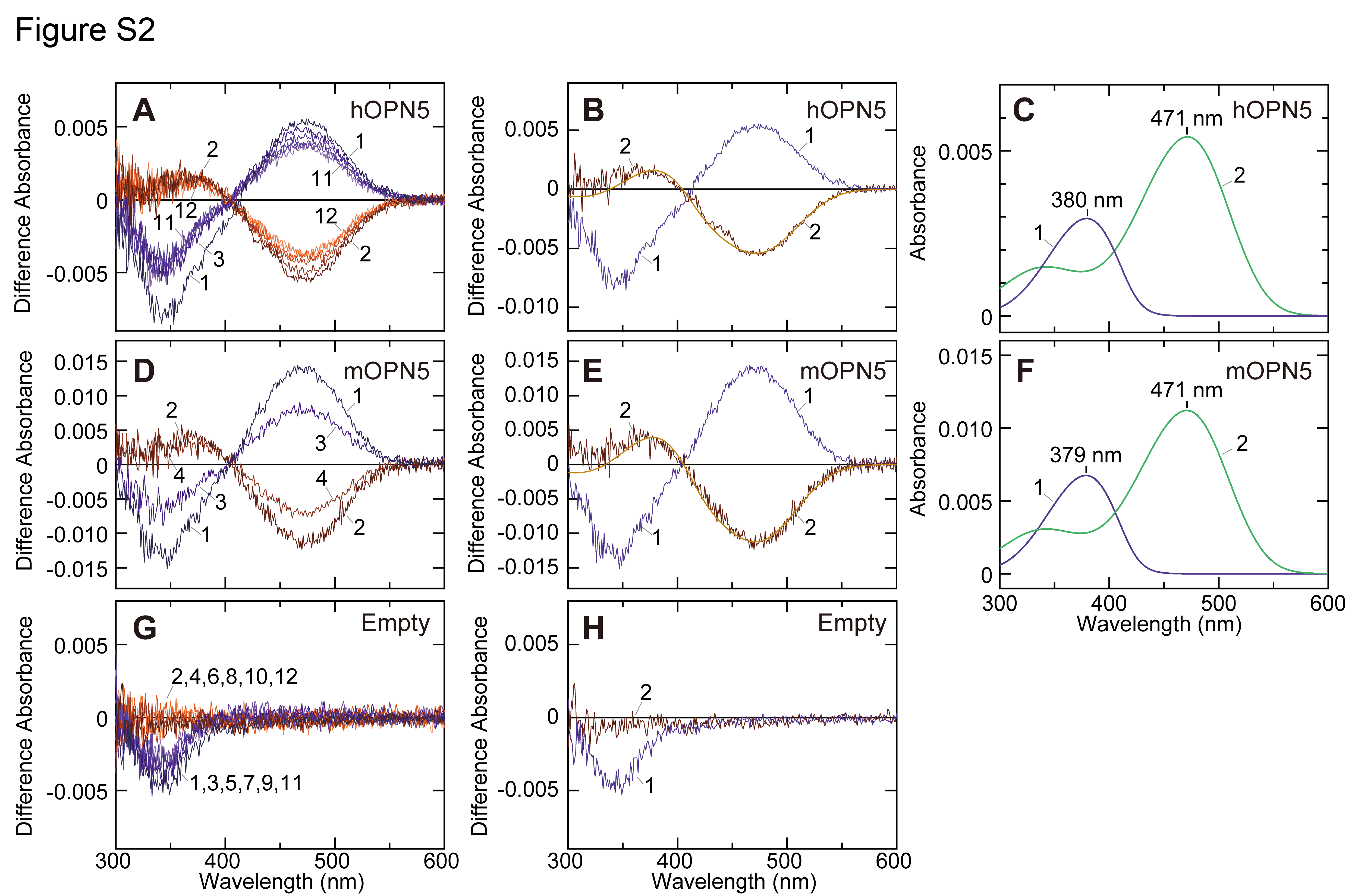

Supplement: Figure S2 — Inference of absorption spectrum of human OPN5. (A) Spectral changes of recombinant human OPN5 during irradiations. HEK293S cells stably expressing human OPN5 (HEK293S-hOPN5#48) were cultured with 11-cis-retinal supplemented, and partially purified into OPN5-containing membrane by sucrose density gradient centrifugation. From this membrane sample, the OPN5 protein were solubilized with 1% DM in buffer P and subjected to spectral measurement. The OPN5 solution was first irradiated with 357-nm UV light (80 µW/cm2) for 4 min, and second with >480-nm yellow light (3.6 mW/cm2, given that it was 550-nm monochromatic light) for 4 min, both with a 24-W metal-halide lamp. Difference spectra were obtained by subtracting the spectrum measured at the first dark state from the one measured after UV irradiation (curve 1) and by subtracting the one after UV irradiation from the one after the yellow irradiation (curve 2). Similar spectral changes were obtained in subsequent repeated irradiations alternately with UV and yellow light (curves 3–12) with gradually decreasing amplitude. (B, C) Inference of human OPN5 spectrum. The difference spectrum between human OPN5 and its photoproduct (curves 2 in panel A and B) was used for the template fitting: First, 450–600 nm region of this difference spectrum (curve 2 in panel B) was best fit to a template for opsin-type photopigments [12] to infer the spectrum of OPN5 photoproduct (curve 2 in panel C). Second, the inferred photoproduct spectrum, peaking at 471 nm, was added to the curve 2 in panel B, to calculate the spectrum corresponding to the dark state OPN5. Finally, this dark state OPN5 spectrum (in the 350–500 nm region) was best fit to the spectral template of opsin-type photopigments, obtaining the curve 1 in panel C. A smooth curve in panel B (superimposed to curve 2) is the reconstructed difference spectrum between inferred OPN5 (curve 1 in panel C) and its photoproduct (curve 2 in panel C) to confirm the validity of the fitting. [file pone.0026388.s002.tif]

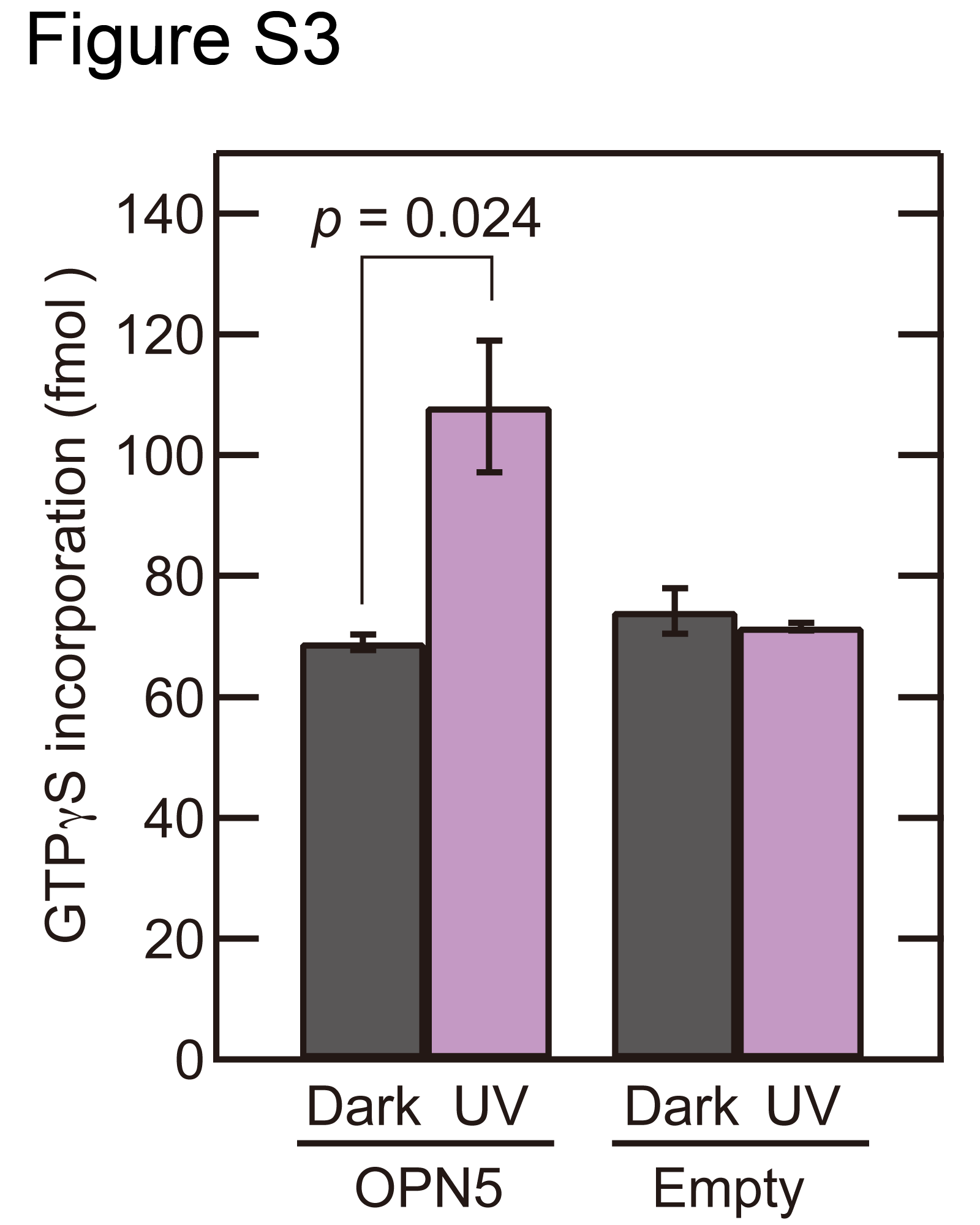

Supplement: Figure S3 — UV-dependent activation of G protein by OPN5. The light-evoked activations of G proteins endogenously expressed in HEK293T/17 cells were measured by a GTPγS-binding assay. The OPN5-containing membrane was fractionated from the HEK293T/17 cells transfected with the expression construct for mouse OPN5 (or the empty vector). The prepared membrane was irradiated with UV-light (357 nm; 14 µW/cm2) for 1 min, and supplied with [S] GTPγS. The incorporated GTPγS during 15 sec of the reaction was quantified. The UV-dependent induction of GTPγS binding activity was detected only in the OPN5-containing membrane with a statistical significance (p = 0.024, two-tailed Student's t-test). Note that no exogenous G protein was supplied into the mixture, indicating the observed GTPγS binding stemmed from the intrinsic activity in the HEK293T/17 cells. All the data were represented by the mean ± SEM (n = 3). (TIF) [file pone.0026388.s003.tif]

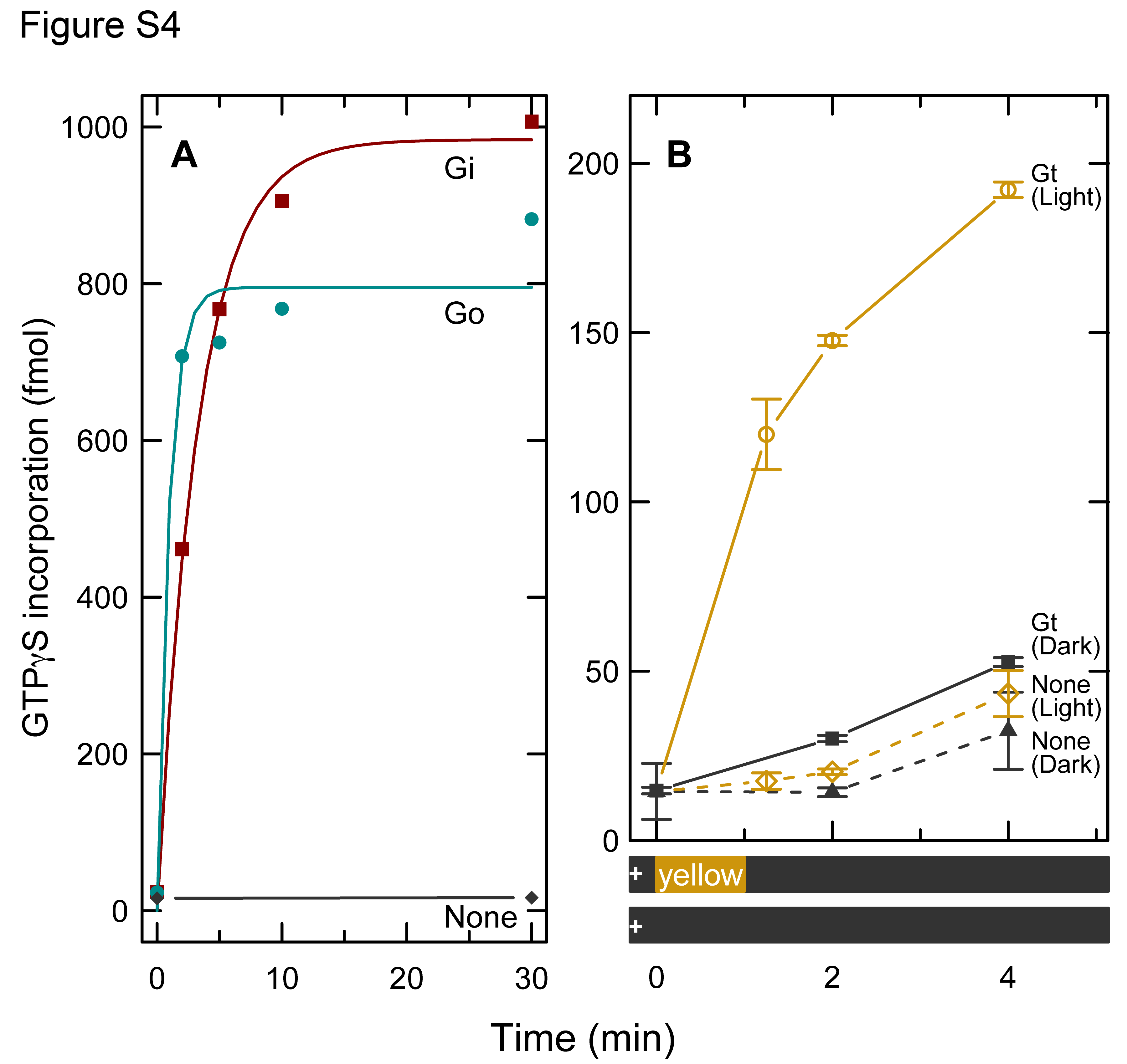

Supplement: Figure S4 — Mg2+- or rhodopsin-induced activity of the G proteins. (A) Mg2+-induced intrinsic activity of Gi and Go. Reaction mixture containing Gi (squares), Go (circles) or no G protein (diamonds) was incubated with [35S] GTPγS in the presence of high concentration of Mg2+ ion at 30°C to measure the maximal activity of GTPγS binding. Buffer composition of the reaction mixture was 20 mM Tris-HCl (pH 7.5), 20 mM MgCl2, 250 mM (NH4)2SO4, 1 mM EDTA, 1 mM DTT, 0.01% Lubrol-PX, 2 µg/µL ovalbumin, 0.1 µM GDP, 1 µM [35S] GTPγS, 0.1 µM heterotrimeric G protein (Gi or Go). After the mixture was incubated for a selected amount of time, its aliquot (10 µL) was mixed with 100 µL of stop solution and immediately filtrated with nitrocellulose membrane (Millipore) to trap [35S] GTPγS bound to G proteins, as described in the Materials and Methods section. (B) GTPγS binding activity of Gt induced by photo-activated rhodopsin. The HEK293T/17 membrane containing bovine rhodopsin was prepared in a similar way for mouse OPN5. The membrane was mixed with Gt or no G protein and then irradiated with yellow light (>490 nm, Light) with the light intensity of 10 mW/cm2 (given that it was 550-nm monochromatic light), or kept in the dark (Dark) for 1 min. Buffer composition of reaction mixture was the same as that for Figure 2 except for absence of GDP in this experiment. The incorporated GTPγS was quantified at each time point. Data were represented by the mean ± SEM (n = 3). (TIF) [file pone.0026388.s004.tif]

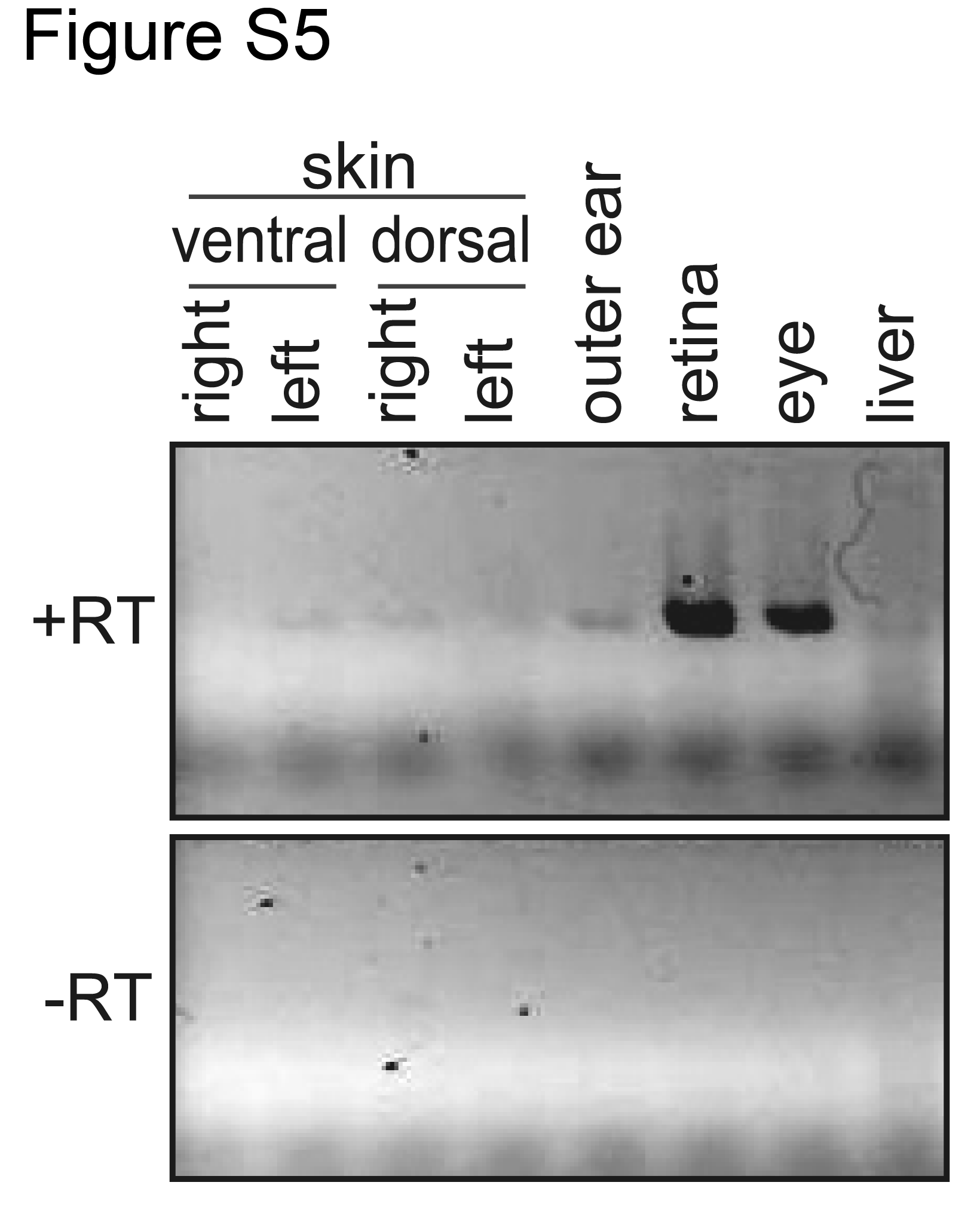

Supplement: Figure S5 — Opn5 mRNA expression in mouse tissues examined by RT-PCR. RNA from mouse tissues (ventral and dorsal skin, outer ear, retina, eye and liver) was subjected to RT-PCR experiment with (+RT) or without (–RT) reverse transcriptase. The 503-bp DNA fragment derived from Opn5 mRNA (arrowhead) was only detected in “+RT” samples but not in “–RT” samples. (TIF) [file pone.0026388.s005.tif]

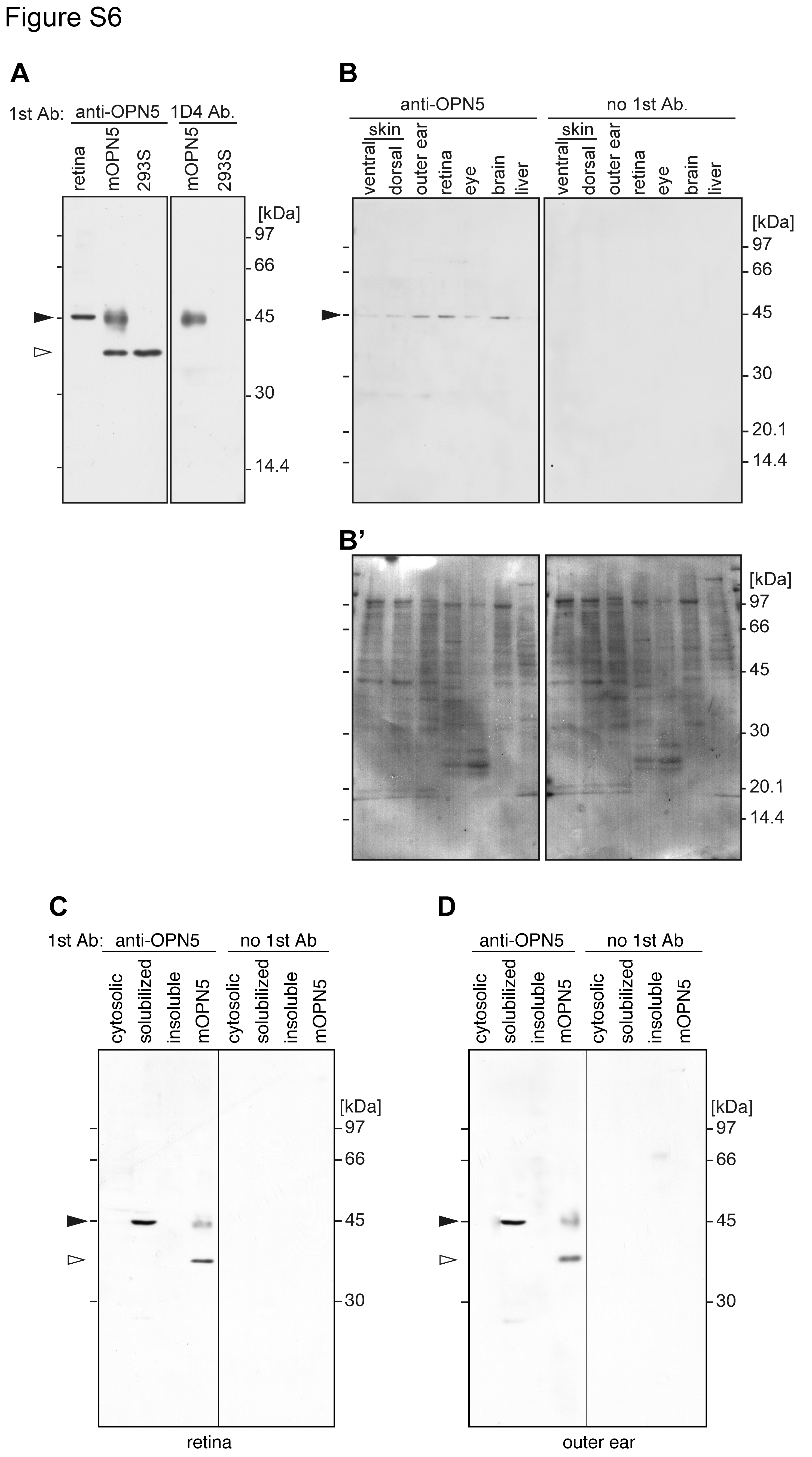

Supplement: Figure S6 — Immunoblot analysis of OPN5 in the mouse tissue extracts. (A) Examination of OPN5 antibody specificity. Loaded are the detergent-extract of membrane fraction of mouse retina (retina), mouse OPN5-expressing (mOPN5) and non-transfected HEK293S cells (293S). The membranes were reacted with the anti-OPN5 antibody (left) and with the 1D4 antibody (right). Both antibodies detected the 45 kDa-band (solid arrowhead) in mOPN5, demonstrating that the anti-OPN5 antibody recognizes the (1D4-epitope-tagged) OPN5 protein. In the mouse retina, the anti-OPN5 antibody detected a single band having a similar size (45 kDa), which should be the mouse native OPN5 protein. In the mOPN5, this antibody detected another band (36 kDa, open arrowhead), which is likely to be an endogenous protein of the human cell line (HEK293S) as it was also detected in the parent 293S by the anti-OPN5 antibody. Importantly, the 36-kDa band was undetectable in any of the mouse tissues tested (see also B–D). (B) Tissue distribution pattern of OPN5 protein expression in mice. Loaded were the tissue extracts containing 10 µg of total proteins from ventral skin, dorsal skin, outer ear, retina, eye, brain, and liver. The blotted membrane was reacted with the anti-OPN5 antibody (left) to visualize OPN5 expression, or with depletion of the primary antibody (right). Note that only a single 45-kDa band (closed arrowhead) was detected with the anti-OPN5 antibody (left). As a loading control (B′), the proteins blotted on these membranes were visualized with MemCode Reversible Protein Stain Kit (Pierce/Thermo Scientific). (C, D) OPN5 localization in the tissue fractions from mouse retina (C) and outer ears (D). The cytosolic, detergent-solubilized and insoluble fractions from these tissues (see Materials and Methods for details) were loaded as well as the detergent-solubilized membrane fraction of mouse OPN5-expressing HEK293S cells (mOPN5, as a positive control). The blotted membrane was reacted with the anti-OPN5 anti [file pone.0026388.s006.tif]

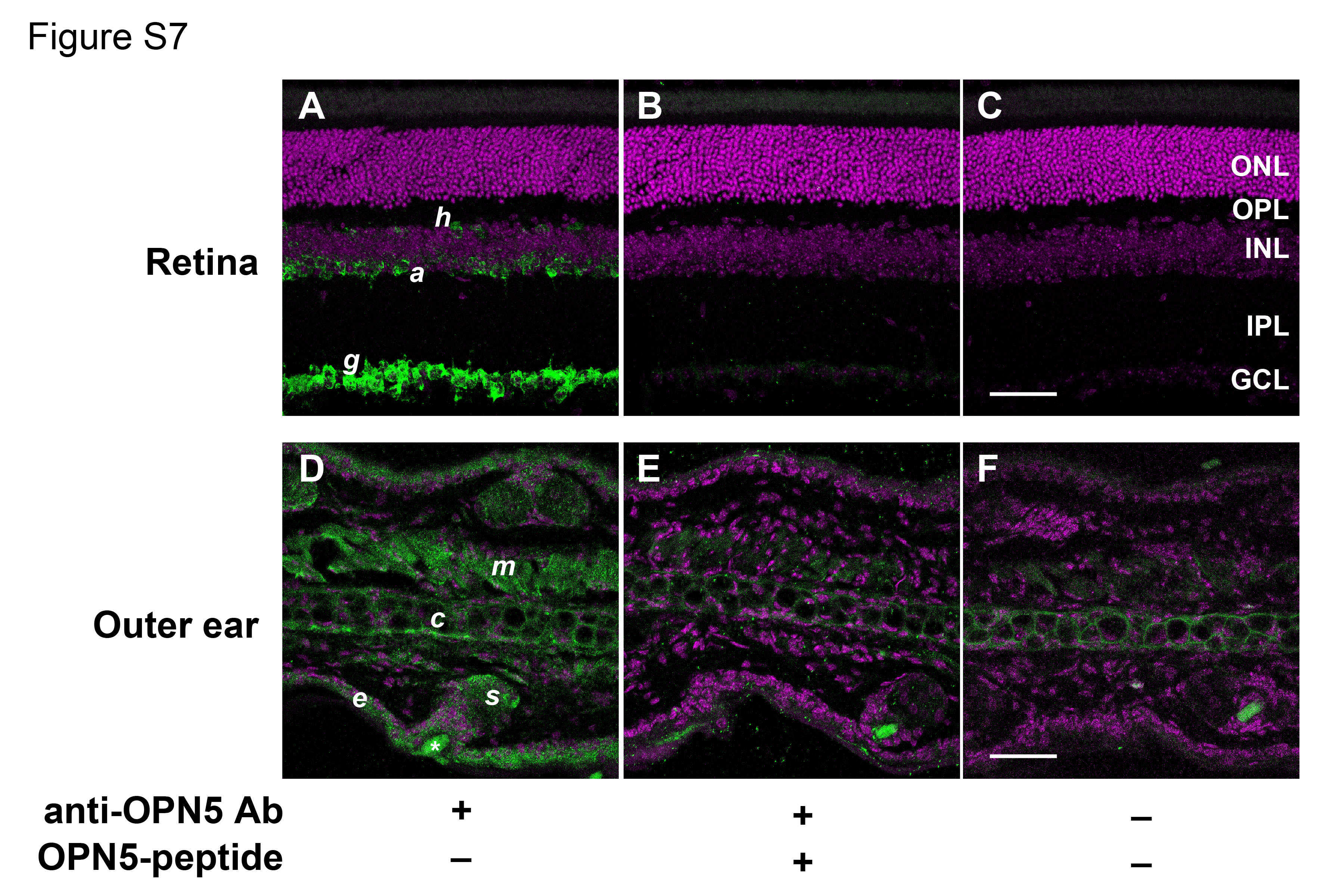

Supplement: Figure S7 — Peptide-preadsorption experiments to examine specificity of immunofluorecent detection of OPN5 in the frozen sections from mouse retina (A–C) and outer ears (D–F). The sections were immuno-reacted with the OPN5 antibody (A, D), or with the antibody that was pre-incubated with ten-fold molar excess of its antigenic peptide (B, E). The sections in C and F were immuno-reacted with depletion of the primary antibody (the secondary antibody alone). Immuno-reactive signals (green) are shown with nuclear staining by TO-PRO-3 (magenta). The peptide-preadsorption treatment (B, E) strongly suppressed the immuno-reactive signals in the ganglion cells (g), the amacrine cells (a) and the horizontal cells (h) in the retina as well as those in the epidermis (e), the striated muscle (m) and the sebaceous gland (s) in the outer ears. Note that in the outer ears (D–F), the hairs (*) exhibit autofluorescence while cartilage (c) has non-specific binding of the secondary antibody. ONL, outer nuclear layer; OPL, outer plexiform layer; INL, inner nuclear layer; IPL, inner plexiform layer; GCL, ganglion cell layer. Scale bars, 50 µm. (TIF) [file pone.0026388.s007.tif]

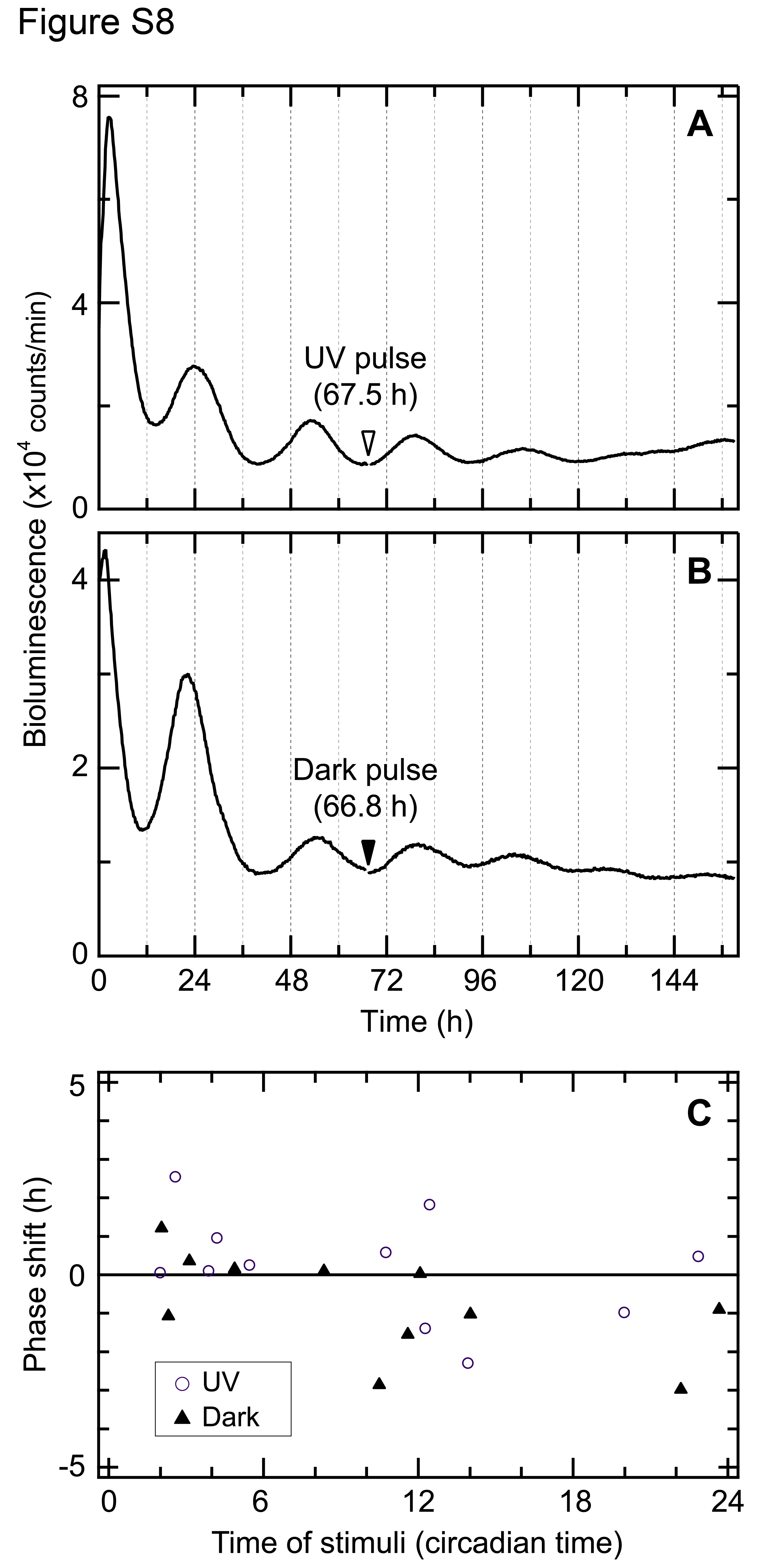

Supplement: Figure S8 — UV irradiation on isolated outer ears of PER2::LUC mice causes no detectable effect on circadian expression of PER2::LUC bioluminescence. (A) Plot of PER2::LUC bioluminescence from a cultured slice of outer ear, showing a circadian rhythm. The sample was subjected to a 30-min pulse of UV light (UV pulse; 357 nm, 11 µW/cm2) from 67.5 h after the initiation of the bioluminescence measurement. This time point (time of stimuli) was calculated as CT 1.99 according to (i) an estimated period for the bioluminescence cycles before the UV pulse and (ii) the third trough as a “datum point” (CT 0). In a similar way, the same time point was calculated as CT 2.05 according to (i) an estimated period for the bioluminescence cycles after the UV pulse and (ii) the fourth trough as a “datum point” (CT 0). From these two different estimates, the phase shift induced by the UV pulse was calculated as 0.06 h in this panel. (B) Plot of PER2::LUC bioluminescence from another cultured slice of outer ear, which was subjected to a 30-min pulse of dark incubation (dark pulse) as a control. (C) Plot of the phase shifts induced by UV-pulse (open circles) and dark-pulse (closed triangles) treatments on the outer ear slices against the time of stimulus. No obvious difference was observed between the phase-shifting effects of the UV-pulse and the dark-pulse treatments. (TIF) [file pone.0026388.s008.tif]

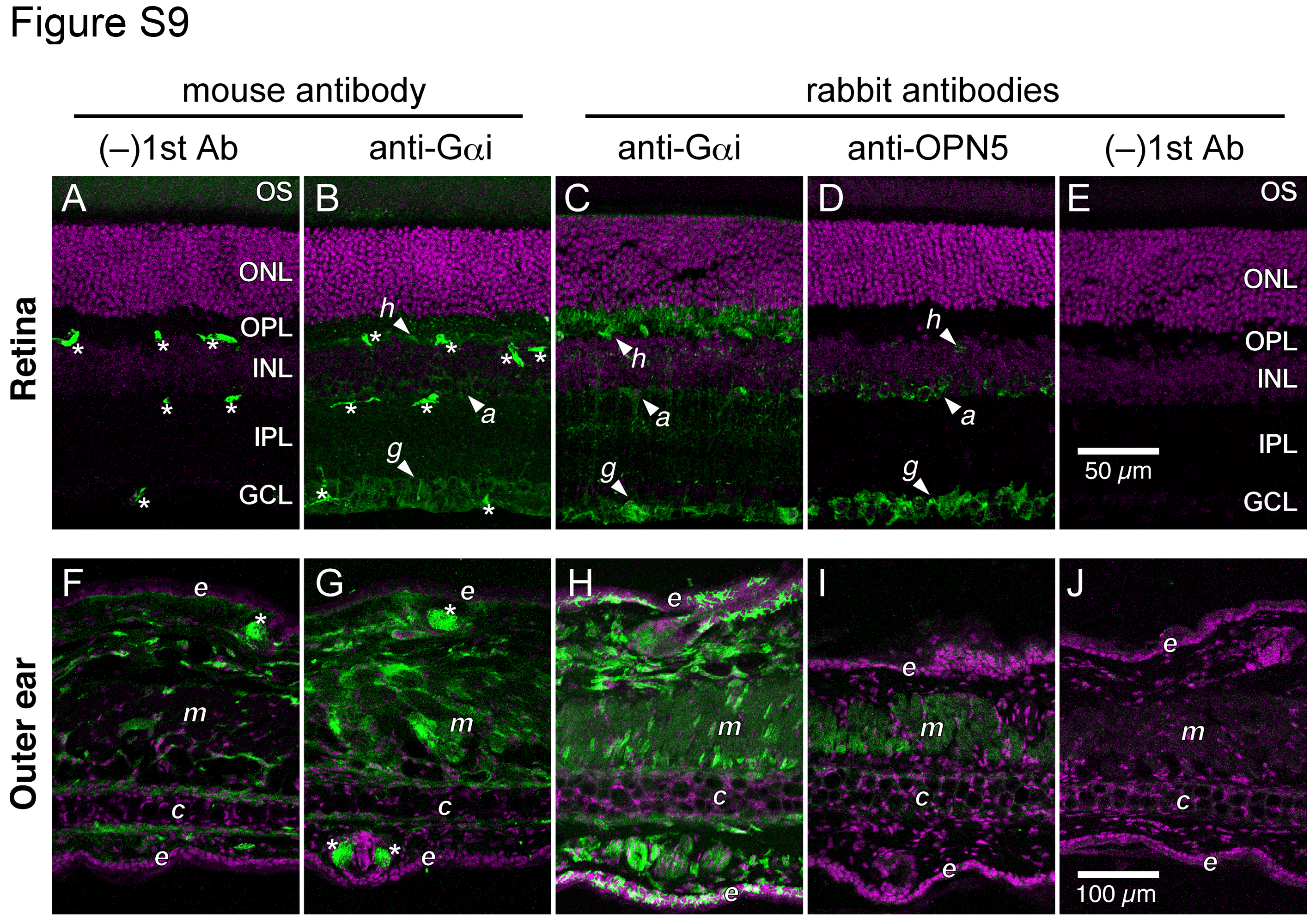

Supplement: Figure S9 — Immunofluorescent examination by anti-Gαi antibodies of the frozen sections from mouse retina (A–E) and outer ears (F–J). The sections were immuno-reacted with mouse anti-Gαi antibody (sc-365422) (B, G), rabbit anti-Gαi antibody [38] (C, H) and rabbit anti-OPN5 antibody (D, I). The sections reacted with the secondary antibody alone (i.e., with depletion of the primary antibody) are shown in A and F as the negative controls for B and G (mouse antibody), and shown in E and J as the negative controls for C–D and H–I (rabbit antibodies). The immuno-reactive signals (green) are shown with nuclear staining by TO-PRO-3 (magenta). In the retinal sections, the immuno-reactive signals were detectable in the horizontal (h), amacrine (a) and ganglion (g) cells by the mouse and the rabbit anti-Gαi antibodies (B, C) as well as the anti-OPN5 antibody (D). In the outer ear sections, the immuno-reactive signals were detectable in the striated muscle cells (m) by the mouse and the rabbit anti-Gαi antibodies (G, H) as well as the anti-OPN5 antibody (I), while the signals in the epidermal cells (e) largely varied in strength among these antibodies. Note that the green signals indicated by asterisks (*) in the panels A, B, G and H originated from direct reaction of the secondary antibody to mouse IgG. OS, outer segments of photoreceptors; ONL, outer nuclear layer; OPL, outer plexiform layer; INL, inner nuclear layer; IPL, inner plexiform layer; GCL, ganglion cell layer; c, cartilage. (TIF) [file pone.0026388.s009.tif]
